# Supplementary figures and images for: Identification of natural antiviral drug candidates against Tilapia Lake Virus: Computational drug design approaches
Source: PLoS One. 2023 Nov 8;18(11):e0287944. doi: 10.1371/journal.pone.0287944 (PMC10631680; doi:10.1371/journal.pone.0287944)

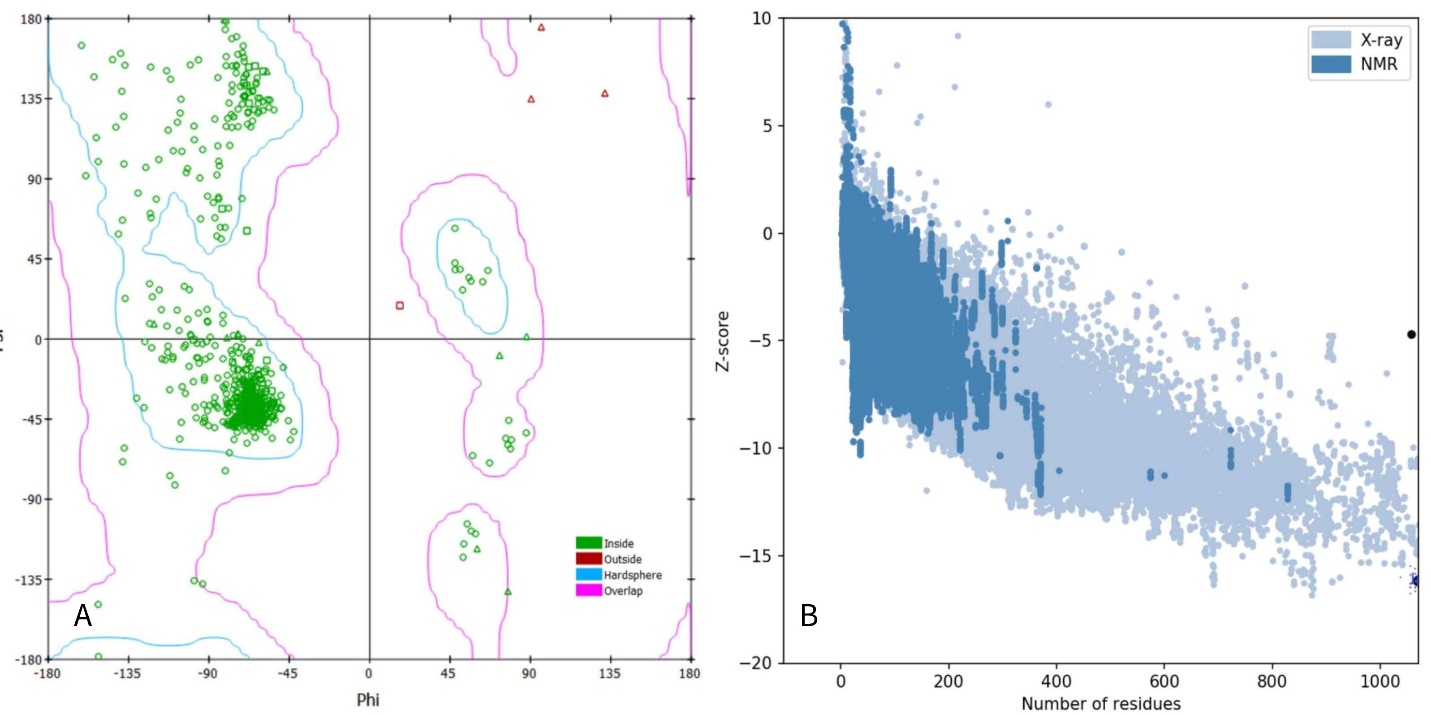

Supplement: S1 Fig — is included to show validation of the 3D structure of the CRM1 protein. (A) The Ramachandran plot statistics represent the most favorable, accepted, a disallowed region with a percentage of 97.984, 2.016, and 0.000%, respectively, and (B) the Z-score of refine gag protein −4.85. (DOCX) [file pone.0287944.s001.docx]

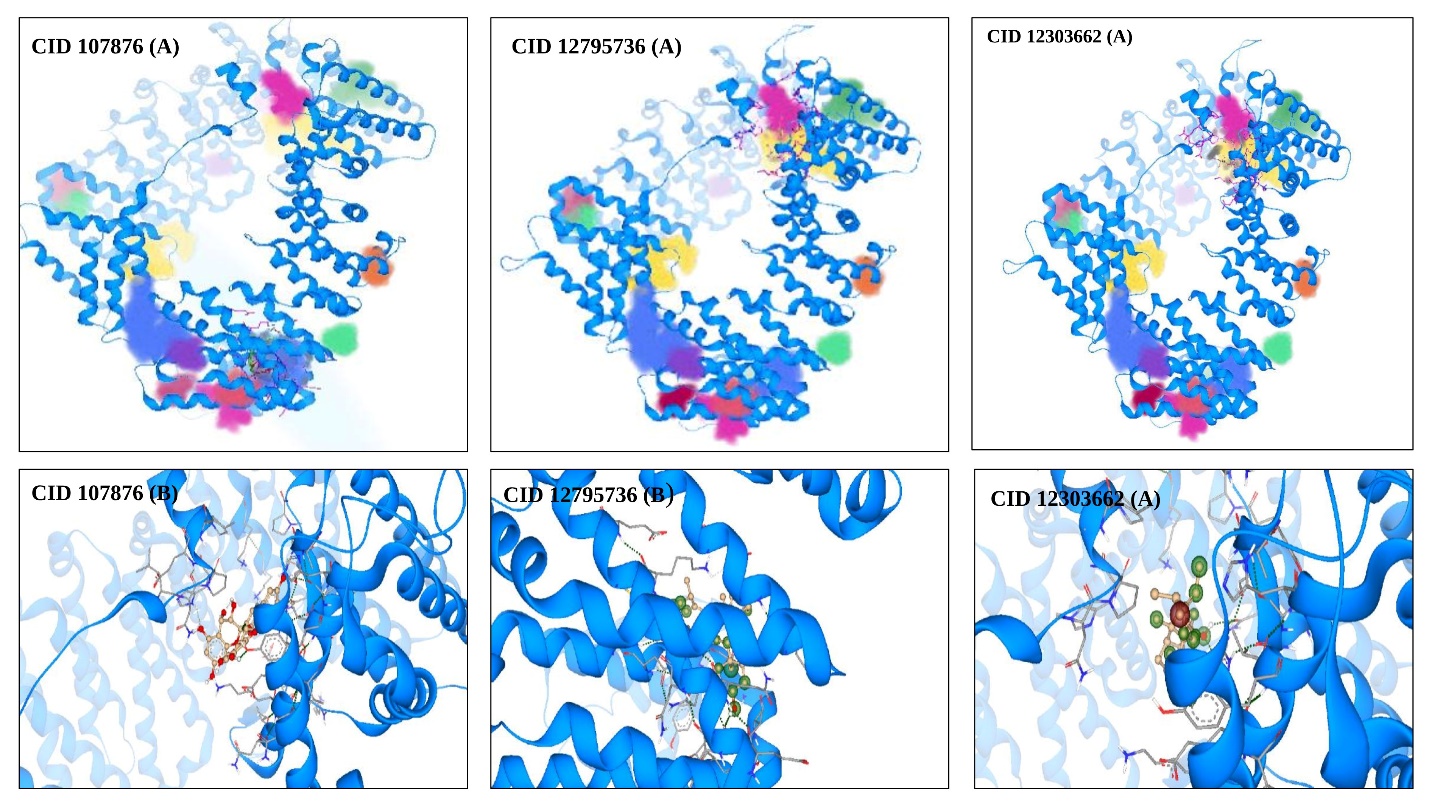

Supplement: S2 Fig — Illustrations of the three selected compounds and their interactions and active poses with protein after re-docking. (DOCX) [file pone.0287944.s002.docx]

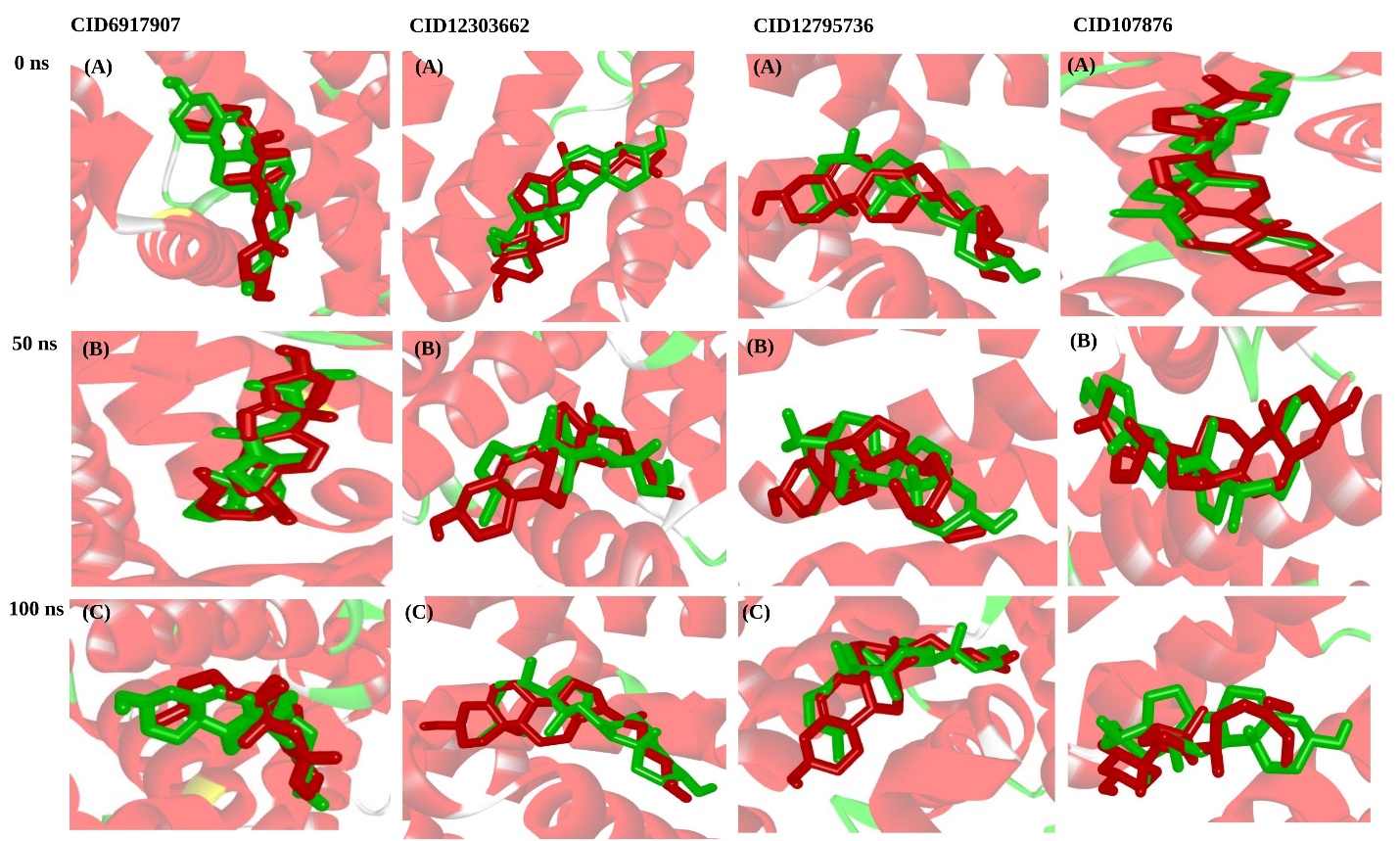

Supplement: S3 Fig — RMSDs are expressed in units of nanoseconds (ns) in this analysis. The ligand-hiMGAM snapshots of three phytochemical substances (Phytosterols, Avenasterol, and Procyanidin) and a marketable medication (Leptomycin B) were overlaid at the start and end timeframes. The ligands and bound hiMGAM proteins are visually distinguished by the colours green and red, respectively, in relation to the extracted frames at 0 ns and 100 ns. (DOCX) [file pone.0287944.s003.docx]
