# Supplementary material for: Identification of natural antiviral drug candidates against Tilapia Lake Virus: Computational drug design approaches
Source: PLoS One. 2023 Nov 8;18(11):e0287944. doi: 10.1371/journal.pone.0287944 (PMC10631680; doi:10.1371/journal.pone.0287944)
Supplement: S1 Table — Calculated locations of CRM1 binding and interactions with each of the three selected proteins. (DOCX) [file pone.0287944.s004.docx]

|  | | | | |
| --- | --- | --- | --- | --- |
| A | 1 | MET | N |  |
| 1 | A | 1 | MET | SD |
| 1 | A | 1 | MET | CE |
| 1 | A | 1 | MET | O |
| 1 | A | 31 | VAL | CG1 |
| 1 | A | 34 | CYS | O |
| 1 | A | 35 | LEU | CB |
| 1 | A | 35 | LEU | CD2 |
| 1 | A | 38 | GLY | CA |
| 1 | A | 38 | GLY | O |
| 1 | A | 41 | PRO | CA |
| 1 | A | 41 | PRO | CB |
| 1 | A | 41 | PRO | CG |
| 1 | A | 43 | GLN | CB |
| 1 | A | 43 | GLN | C |
| 1 | A | 44 | ARG | N |
| 1 | A | 44 | ARG | CA |
| 1 | A | 44 | ARG | CB |
| 1 | A | 44 | ARG | CG |
| 1 | A | 44 | ARG | CD |
| 1 | A | 44 | ARG | NE |
| 1 | A | 44 | ARG | CZ |
| 1 | A | 44 | ARG | NH1 |
| 1 | A | 44 | ARG | NH2 |
| 1 | A | 44 | ARG | O |
| 1 | A | 47 | GLN | CB |
| 1 | A | 47 | GLN | CG |
| 1 | A | 47 | GLN | CD |
| 1 | A | 47 | GLN | OE1 |
| 1 | A | 47 | GLN | NE2 |
| 1 | A | 47 | GLN | C |
| 1 | A | 47 | GLN | O |
| 1 | A | 48 | GLU | N |
| 1 | A | 48 | GLU | CA |
| 1 | A | 48 | GLU | CB |
| 1 | A | 48 | GLU | CG |
| 1 | A | 48 | GLU | CD |
| 1 | A | 48 | GLU | OE1 |
| 1 | A | 48 | GLU | OE2 |
| 1 | A | 50 | LEU | CD2 |
| 1 | A | 51 | THR | CB |
| 1 | A | 51 | THR | CG2 |
| 1 | A | 51 | THR | OG1 |
| 1 | A | 52 | HIS | CE1 |
| 1 | A | 54 | LYS | CD |
| 1 | A | 54 | LYS | CE |
| 1 | A | 54 | LYS | NZ |
| 1 | A | 77 | TYR | CE1 |
| 1 | A | 77 | TYR | OH |
| 1 | A | 77 | TYR | CE2 |
| 1 | A | 78 | TYR | CE1 |
| 1 | A | 78 | TYR | CZ |
| 1 | A | 78 | TYR | OH |
| 1 | A | 78 | TYR | CE2 |
| 1 | A | 81 | GLN | CB |
| 1 | A | 81 | GLN | CG |
| 1 | A | 81 | GLN | CD |
| 1 | A | 81 | GLN | OE1 |
| 1 | A | 81 | GLN | NE2 |
| 1 | A | 81 | GLN | C |
| 1 | A | 81 | GLN | O |
| 1 | A | 82 | ILE | CA |
| 1 | A | 84 | GLU | CB |
| 1 | A | 84 | GLU | CG |
| 1 | A | 84 | GLU | CD |
| 1 | A | 84 | GLU | OE1 |
| 1 | A | 84 | GLU | OE2 |
| 1 | A | 84 | GLU | C |
| 1 | A | 84 | GLU | O |
| 1 | A | 85 | THR | N |
| 1 | A | 85 | THR | CA |
| 1 | A | 85 | THR | CG2 |
| 1 | A | 85 | THR | OG1 |
| 1 | A | 87 | ILE | CG2 |
| 1 | A | 87 | ILE | O |
| 1 | A | 88 | LYS | CA |
| 1 | A | 88 | LYS | CB |
| 1 | A | 88 | LYS | CG |
| 1 | A | 88 | LYS | CD |
| 1 | A | 88 | LYS | CE |
| 1 | A | 88 | LYS | NZ |
| 1 | A | 88 | LYS | O |
| 1 | A | 89 | THR | CA |
| 1 | A | 89 | THR | OG1 |
| 1 | A | 92 | LYS | CG |
| 1 | A | 92 | LYS | CE |
| 1 | A | 92 | LYS | NZ |
| 1 | A | 114 | SER | CA |
| 1 | A | 114 | SER | CB |
| 1 | A | 120 | VAL | CG1 |
| 1 | A | 120 | VAL | CG2 |
| 1 | A | 124 | LYS | CA |
| 1 | A | 124 | LYS | CB |
| 1 | A | 124 | LYS | CG |
| 1 | A | 124 | LYS | CD |
| 1 | A | 124 | LYS | CE |
| 1 | A | 124 | LYS | NZ |
| 1 | A | 124 | LYS | C |
| 1 | A | 124 | LYS | O |
| 1 | A | 125 | VAL | N |
| 1 | A | 125 | VAL | CA |
| 1 | A | 125 | VAL | CG1 |
| 1 | A | 125 | VAL | CG2 |
| 1 | A | 125 | VAL | O |
| 1 | A | 127 | ILE | CB |
| 1 | A | 127 | ILE | CG2 |
| 1 | A | 127 | ILE | CD1 |
| 1 | A | 128 | GLY | N |
| 1 | A | 128 | GLY | CA |
| 1 | A | 128 | GLY | C |
| 1 | A | 128 | GLY | O |
| 1 | A | 129 | LYS | N |
| 1 | A | 129 | LYS | CA |
| 1 | A | 129 | LYS | CG |
| 1 | A | 129 | LYS | CD |
| 1 | A | 129 | LYS | CE |
| 1 | A | 129 | LYS | NZ |
| 1 | A | 131 | ASN | ND2 |
| 1 | A | 132 | MET | CB |
| 1 | A | 132 | MET | CG |
| 1 | A | 132 | MET | SD |
| 1 | A | 132 | MET | CE |
| 1 | A | 136 | GLN | CB |
| 1 | A | 136 | GLN | CG |
| 1 | A | 136 | GLN | CD |
| 1 | A | 136 | GLN | OE1 |
| 1 | A | 136 | GLN | NE2 |
| 1 | A | 136 | GLN | O |
| 1 | A | 139 | LYS | CB |
| 1 | A | 139 | LYS | CG |
| 1 | A | 139 | LYS | CD |
| 1 | A | 139 | LYS | CE |
| 1 | A | 139 | LYS | NZ |
| 1 | A | 139 | LYS | O |
| 1 | A | 140 | GLN | N |
| 1 | A | 140 | GLN | CA |
| 1 | A | 140 | GLN | CB |
| 1 | A | 140 | GLN | CD |
| 1 | A | 140 | GLN | OE1 |
| 1 | A | 140 | GLN | NE2 |
| 1 | A | 140 | GLN | C |
| 1 | A | 140 | GLN | O |
| 1 | A | 141 | GLU | CA |
| 1 | A | 141 | GLU | CG |
| 1 | A | 143 | PRO | CG |
| 1 | A | 143 | PRO | CD |
| 1 | A | 144 | LYS | CB |
| 1 | A | 144 | LYS | CG |
| 1 | A | 144 | LYS | CD |
| 1 | A | 144 | LYS | CE |
| 1 | A | 144 | LYS | NZ |
| 1 | A | 145 | HIS | ND1 |
| 1 | A | 145 | HIS | CE1 |
| 1 | A | 162 | SER | CA |
| 1 | A | 162 | SER | CB |
| 1 | A | 162 | SER | O |
| 1 | A | 165 | GLN | CB |
| 1 | A | 165 | GLN | CD |
| 1 | A | 165 | GLN | OE1 |
| 1 | A | 165 | GLN | NE2 |
| 1 | A | 165 | GLN | C |
| 1 | A | 166 | ASN | N |
| 1 | A | 166 | ASN | CA |
| 1 | A | 166 | ASN | CB |
| 1 | A | 166 | ASN | OD1 |
| 1 | A | 169 | VAL | CB |
| 1 | A | 169 | VAL | CG1 |
| 1 | A | 169 | VAL | CG2 |
| 1 | A | 172 | LYS | CE |
| 1 | A | 172 | LYS | NZ |
| 1 | A | 173 | LEU | CB |
| 1 | A | 173 | LEU | CD1 |
| 1 | A | 173 | LEU | CD2 |
| 1 | A | 173 | LEU | O |
| 1 | A | 176 | GLU | CB |
| 1 | A | 176 | GLU | CG |
| 1 | A | 176 | GLU | CD |
| 1 | A | 176 | GLU | OE1 |
| 1 | A | 176 | GLU | OE2 |
| 1 | A | 177 | GLU | CG |
| 1 | A | 177 | GLU | CD |
| 1 | A | 177 | GLU | OE1 |
| 1 | A | 177 | GLU | OE2 |
| 1 | A | 178 | VAL | O |
| 1 | A | 179 | PHE | CA |
| 1 | A | 179 | PHE | CB |
| 1 | A | 179 | PHE | CE2 |
| 1 | A | 179 | PHE | CD2 |
| 1 | A | 179 | PHE | C |
| 1 | A | 179 | PHE | O |
| 1 | A | 180 | ASP | CA |
| 1 | A | 180 | ASP | CB |
| 1 | A | 180 | ASP | CG |
| 1 | A | 180 | ASP | OD1 |
| 1 | A | 180 | ASP | OD2 |
| 1 | A | 180 | ASP | C |
| 1 | A | 180 | ASP | O |
| 1 | A | 181 | PHE | CA |
| 1 | A | 181 | PHE | CB |
| 1 | A | 181 | PHE | CD1 |
| 1 | A | 181 | PHE | CE1 |
| 1 | A | 181 | PHE | CZ |
| 1 | A | 181 | PHE | CE2 |
| 1 | A | 181 | PHE | CD2 |
| 1 | A | 181 | PHE | O |
| 1 | A | 182 | SER | CB |
| 1 | A | 182 | SER | O |
| 1 | A | 183 | SER | N |
| 1 | A | 183 | SER | CA |
| 1 | A | 183 | SER | CB |
| 1 | A | 183 | SER | OG |
| 1 | A | 183 | SER | C |
| 1 | A | 183 | SER | O |
| 1 | A | 184 | GLY | N |
| 1 | A | 184 | GLY | CA |
| 1 | A | 184 | GLY | O |
| 1 | A | 185 | GLN | CA |
| 1 | A | 185 | GLN | CG |
| 1 | A | 185 | GLN | CD |
| 1 | A | 185 | GLN | OE1 |
| 1 | A | 185 | GLN | NE2 |
| 1 | A | 185 | GLN | O |
| 1 | A | 186 | MET | CG |
| 1 | A | 186 | MET | SD |
| 1 | A | 186 | MET | O |
| 1 | A | 187 | THR | CG2 |
| 1 | A | 188 | GLN | N |
| 1 | A | 188 | GLN | CA |
| 1 | A | 188 | GLN | CB |
| 1 | A | 188 | GLN | CG |
| 1 | A | 188 | GLN | CD |
| 1 | A | 188 | GLN | OE1 |
| 1 | A | 188 | GLN | NE2 |
| 1 | A | 188 | GLN | O |
| 1 | A | 189 | VAL | CA |
| 1 | A | 189 | VAL | CG1 |
| 1 | A | 189 | VAL | CG2 |
| 1 | A | 189 | VAL | O |
| 1 | A | 190 | LYS | CA |
| 1 | A | 190 | LYS | CB |
| 1 | A | 190 | LYS | CG |
| 1 | A | 190 | LYS | CD |
| 1 | A | 190 | LYS | CE |
| 1 | A | 190 | LYS | NZ |
| 1 | A | 190 | LYS | O |
| 1 | A | 191 | ALA | CB |
| 1 | A | 191 | ALA | C |
| 1 | A | 191 | ALA | O |
| 1 | A | 192 | LYS | N |
| 1 | A | 192 | LYS | CA |
| 1 | A | 192 | LYS | CB |
| 1 | A | 192 | LYS | CG |
| 1 | A | 192 | LYS | CD |
| 1 | A | 192 | LYS | CE |
| 1 | A | 192 | LYS | NZ |
| 1 | A | 193 | HIS | N |
| 1 | A | 193 | HIS | CA |
| 1 | A | 193 | HIS | CB |
| 1 | A | 193 | HIS | CG |
| 1 | A | 193 | HIS | ND1 |
| 1 | A | 193 | HIS | CE1 |
| 1 | A | 193 | HIS | CD2 |
| 1 | A | 194 | LEU | CG |
| 1 | A | 194 | LEU | CD1 |
| 1 | A | 194 | LEU | CD2 |
| 1 | A | 195 | LYS | CB |
| 1 | A | 195 | LYS | CD |
| 1 | A | 195 | LYS | CE |
| 1 | A | 195 | LYS | NZ |
| 1 | A | 199 | CYS | SG |
| 1 | A | 220 | PRO | CA |
| 1 | A | 220 | PRO | CB |
| 1 | A | 220 | PRO | O |
| 1 | A | 223 | HIS | CB |
| 1 | A | 223 | HIS | CE1 |
| 1 | A | 223 | HIS | NE2 |
| 1 | A | 223 | HIS | CD2 |
| 1 | A | 224 | ALA | N |
| 1 | A | 224 | ALA | CA |
| 1 | A | 224 | ALA | CB |
| 1 | A | 227 | GLU | CG |
| 1 | A | 227 | GLU | CD |
| 1 | A | 227 | GLU | OE1 |
| 1 | A | 227 | GLU | OE2 |
| 1 | A | 230 | LEU | CG |
| 1 | A | 230 | LEU | CD1 |
| 1 | A | 230 | LEU | CD2 |
| 1 | A | 230 | LEU | O |
| 1 | A | 231 | ARG | CA |
| 1 | A | 231 | ARG | CD |
| 1 | A | 231 | ARG | NE |
| 1 | A | 231 | ARG | CZ |
| 1 | A | 231 | ARG | NH1 |
| 1 | A | 231 | ARG | NH2 |
| 1 | A | 233 | LEU | O |
| 1 | A | 234 | ASN | CA |
| 1 | A | 234 | ASN | CB |
| 1 | A | 234 | ASN | CG |
| 1 | A | 234 | ASN | OD1 |
| 1 | A | 234 | ASN | ND2 |
| 1 | A | 234 | ASN | C |
| 1 | A | 234 | ASN | O |
| 1 | A | 235 | TRP | CA |
| 1 | A | 235 | TRP | CB |
| 1 | A | 235 | TRP | C |
| 1 | A | 235 | TRP | O |
| 1 | A | 236 | ILE | N |
| 1 | A | 236 | ILE | O |
| 1 | A | 237 | PRO | CA |
| 1 | A | 237 | PRO | CB |
| 1 | A | 237 | PRO | CG |
| 1 | A | 237 | PRO | CD |
| 1 | A | 238 | LEU | CG |
| 1 | A | 238 | LEU | CD1 |
| 1 | A | 238 | LEU | CD2 |
| 1 | A | 241 | ILE | CD1 |
| 1 | A | 258 | PRO | O |
| 1 | A | 259 | MET | O |
| 1 | A | 262 | ASN | CB |
| 1 | A | 262 | ASN | ND2 |
| 1 | A | 263 | VAL | CG2 |
| 1 | A | 265 | LEU | CB |
| 1 | A | 265 | LEU | C |
| 1 | A | 266 | LYS | CA |
| 1 | A | 266 | LYS | CB |
| 1 | A | 266 | LYS | CG |
| 1 | A | 266 | LYS | CD |
| 1 | A | 266 | LYS | CE |
| 1 | A | 266 | LYS | NZ |
| 1 | A | 266 | LYS | O |
| 1 | A | 269 | THR | CB |
| 1 | A | 269 | THR | CG2 |
| 1 | A | 269 | THR | OG1 |
| 1 | A | 269 | THR | O |
| 1 | A | 270 | GLU | CA |
| 1 | A | 270 | GLU | CB |
| 1 | A | 270 | GLU | CG |
| 1 | A | 270 | GLU | CD |
| 1 | A | 270 | GLU | OE1 |
| 1 | A | 270 | GLU | OE2 |
| 1 | A | 270 | GLU | O |
| 1 | A | 273 | GLY | CA |
| 1 | A | 273 | GLY | C |
| 1 | A | 273 | GLY | O |
| 1 | A | 274 | VAL | CA |
| 1 | A | 274 | VAL | CG1 |
| 1 | A | 274 | VAL | CG2 |
| 1 | A | 275 | SER | N |
| 1 | A | 275 | SER | CA |
| 1 | A | 275 | SER | CB |
| 1 | A | 275 | SER | OG |
| 1 | A | 308 | TYR | OH |
| 1 | A | 313 | ASP | OD1 |
| 1 | A | 313 | ASP | OD2 |
| 1 | A | 313 | ASP | O |
| 1 | A | 314 | ASP | CA |
| 1 | A | 314 | ASP | OD1 |
| 1 | A | 316 | GLN | CB |
| 1 | A | 316 | GLN | OE1 |
| 1 | A | 317 | ASN | CA |
| 1 | A | 317 | ASN | CB |
| 1 | A | 317 | ASN | CG |
| 1 | A | 317 | ASN | OD1 |
| 1 | A | 317 | ASN | ND2 |
| 1 | A | 317 | ASN | O |
| 1 | A | 320 | GLN | CB |
| 1 | A | 320 | GLN | CG |
| 1 | A | 320 | GLN | CD |
| 1 | A | 320 | GLN | OE1 |
| 1 | A | 320 | GLN | NE2 |
| 1 | A | 321 | ASN | CA |
| 1 | A | 321 | ASN | CB |
| 1 | A | 321 | ASN | OD1 |
| 1 | A | 321 | ASN | ND2 |
| 1 | A | 321 | ASN | O |
| 1 | A | 324 | LEU | CA |
| 1 | A | 324 | LEU | CB |
| 1 | A | 324 | LEU | CG |
| 1 | A | 324 | LEU | CD1 |
| 1 | A | 324 | LEU | CD2 |
| 1 | A | 324 | LEU | O |
| 1 | A | 327 | CYS | CB |
| 1 | A | 327 | CYS | C |
| 1 | A | 327 | CYS | O |
| 1 | A | 328 | THR | CA |
| 1 | A | 328 | THR | CG2 |
| 1 | A | 328 | THR | OG1 |
| 1 | A | 331 | LYS | CB |
| 1 | A | 331 | LYS | CG |
| 1 | A | 331 | LYS | CD |
| 1 | A | 331 | LYS | CE |
| 1 | A | 331 | LYS | NZ |
| 1 | A | 331 | LYS | O |
| 1 | A | 332 | GLU | CA |
| 1 | A | 332 | GLU | CG |
| 1 | A | 332 | GLU | CD |
| 1 | A | 332 | GLU | OE1 |
| 1 | A | 332 | GLU | OE2 |
| 1 | A | 362 | GLU | CD |
| 1 | A | 362 | GLU | OE2 |
| 1 | A | 363 | THR | CB |
| 1 | A | 363 | THR | CG2 |
| 1 | A | 364 | GLU | N |
| 1 | A | 364 | GLU | CA |
| 1 | A | 364 | GLU | CB |
| 1 | A | 364 | GLU | CG |
| 1 | A | 364 | GLU | CD |
| 1 | A | 364 | GLU | OE1 |
| 1 | A | 364 | GLU | OE2 |
| 1 | A | 364 | GLU | O |
| 1 | A | 365 | ILE | CG1 |
| 1 | A | 367 | LYS | CA |
| 1 | A | 367 | LYS | CB |
| 1 | A | 367 | LYS | CG |
| 1 | A | 367 | LYS | CD |
| 1 | A | 367 | LYS | CE |
| 1 | A | 367 | LYS | NZ |
| 1 | A | 367 | LYS | C |
| 1 | A | 367 | LYS | O |
| 1 | A | 368 | ILE | CA |
| 1 | A | 368 | ILE | CG2 |
| 1 | A | 368 | ILE | CG1 |
| 1 | A | 368 | ILE | CD1 |
| 1 | A | 368 | ILE | O |
| 1 | A | 370 | LEU | CB |
| 1 | A | 370 | LEU | CD1 |
| 1 | A | 370 | LEU | C |
| 1 | A | 370 | LEU | O |
| 1 | A | 371 | GLU | N |
| 1 | A | 371 | GLU | CA |
| 1 | A | 371 | GLU | CB |
| 1 | A | 371 | GLU | CG |
| 1 | A | 371 | GLU | CD |
| 1 | A | 371 | GLU | OE1 |
| 1 | A | 371 | GLU | OE2 |
| 1 | A | 371 | GLU | O |
| 1 | A | 372 | TYR | N |
| 1 | A | 372 | TYR | CA |
| 1 | A | 372 | TYR | CB |
| 1 | A | 374 | ASN | CB |
| 1 | A | 374 | ASN | CG |
| 1 | A | 374 | ASN | OD1 |
| 1 | A | 374 | ASN | ND2 |
| 1 | A | 375 | HIS | N |
| 1 | A | 375 | HIS | CA |
| 1 | A | 375 | HIS | CB |
| 1 | A | 375 | HIS | ND1 |
| 1 | A | 375 | HIS | CE1 |
| 1 | A | 375 | HIS | O |
| 1 | A | 378 | ALA | CA |
| 1 | A | 378 | ALA | CB |
| 1 | A | 378 | ALA | O |
| 1 | A | 382 | ARG | CG |
| 1 | A | 382 | ARG | CD |
| 1 | A | 382 | ARG | NE |
| 1 | A | 382 | ARG | CZ |
| 1 | A | 382 | ARG | NH1 |
| 1 | A | 382 | ARG | NH2 |
| 1 | A | 426 | LYS | CG |
| 1 | A | 426 | LYS | CD |
| 1 | A | 426 | LYS | CE |
| 1 | A | 426 | LYS | NZ |
| 1 | A | 427 | PRO | CB |
| 1 | A | 427 | PRO | CG |
| 1 | A | 427 | PRO | CD |
| 1 | A | 427 | PRO | O |
| 1 | A | 428 | GLU | N |
| 1 | A | 428 | GLU | CB |
| 1 | A | 428 | GLU | CG |
| 1 | A | 428 | GLU | CD |
| 1 | A | 428 | GLU | OE1 |
| 1 | A | 428 | GLU | OE2 |
| 1 | A | 428 | GLU | O |
| 1 | A | 430 | VAL | CB |
| 1 | A | 430 | VAL | CG1 |
| 1 | A | 430 | VAL | CG2 |
| 1 | A | 430 | VAL | O |
| 1 | A | 431 | LEU | CA |
| 1 | A | 431 | LEU | CD2 |
| 1 | A | 432 | VAL | N |
| 1 | A | 432 | VAL | CG1 |
| 1 | A | 432 | VAL | CG2 |
| 1 | A | 433 | VAL | CG1 |
| 1 | A | 434 | GLU | O |
| 1 | A | 435 | ASN | CA |
| 1 | A | 435 | ASN | CB |
| 1 | A | 435 | ASN | OD1 |
| 1 | A | 435 | ASN | ND2 |
| 1 | A | 435 | ASN | O |
| 1 | A | 436 | ASP | N |
| 1 | A | 436 | ASP | CA |
| 1 | A | 436 | ASP | CB |
| 1 | A | 436 | ASP | CG |
| 1 | A | 436 | ASP | OD1 |
| 1 | A | 436 | ASP | OD2 |
| 1 | A | 436 | ASP | O |
| 1 | A | 437 | GLN | CB |
| 1 | A | 437 | GLN | CG |
| 1 | A | 437 | GLN | NE2 |
| 1 | A | 439 | GLU | CA |
| 1 | A | 439 | GLU | CB |
| 1 | A | 439 | GLU | CG |
| 1 | A | 439 | GLU | CD |
| 1 | A | 439 | GLU | OE1 |
| 1 | A | 439 | GLU | OE2 |
| 1 | A | 440 | VAL | N |
| 1 | A | 440 | VAL | CG1 |
| 1 | A | 440 | VAL | C |
| 1 | A | 440 | VAL | O |
| 1 | A | 441 | VAL | CA |
| 1 | A | 441 | VAL | CG1 |
| 1 | A | 441 | VAL | CG2 |
| 1 | A | 442 | ARG | N |
| 1 | A | 442 | ARG | CA |
| 1 | A | 442 | ARG | CB |
| 1 | A | 442 | ARG | CG |
| 1 | A | 442 | ARG | CD |
| 1 | A | 442 | ARG | NE |
| 1 | A | 442 | ARG | NH1 |
| 1 | A | 442 | ARG | O |
| 1 | A | 443 | GLU | CA |
| 1 | A | 443 | GLU | CG |
| 1 | A | 443 | GLU | CD |
| 1 | A | 443 | GLU | OE1 |
| 1 | A | 443 | GLU | OE2 |
| 1 | A | 444 | PHE | N |
| 1 | A | 444 | PHE | CB |
| 1 | A | 444 | PHE | CD1 |
| 1 | A | 444 | PHE | CE1 |
| 1 | A | 444 | PHE | CZ |
| 1 | A | 444 | PHE | CE2 |
| 1 | A | 444 | PHE | CD2 |
| 1 | A | 444 | PHE | O |
| 1 | A | 445 | MET | CA |
| 1 | A | 445 | MET | SD |
| 1 | A | 445 | MET | CE |
| 1 | A | 446 | LYS | N |
| 1 | A | 446 | LYS | CB |
| 1 | A | 446 | LYS | CG |
| 1 | A | 446 | LYS | CD |
| 1 | A | 446 | LYS | CE |
| 1 | A | 446 | LYS | NZ |
| 1 | A | 446 | LYS | O |
| 1 | A | 447 | ASP | N |
| 1 | A | 447 | ASP | CA |
| 1 | A | 447 | ASP | CB |
| 1 | A | 447 | ASP | CG |
| 1 | A | 447 | ASP | OD1 |
| 1 | A | 447 | ASP | OD2 |
| 1 | A | 447 | ASP | C |
| 1 | A | 447 | ASP | O |
| 1 | A | 448 | THR | CA |
| 1 | A | 448 | THR | CB |
| 1 | A | 448 | THR | CG2 |
| 1 | A | 449 | ASP | N |
| 1 | A | 449 | ASP | CA |
| 1 | A | 449 | ASP | CB |
| 1 | A | 449 | ASP | CG |
| 1 | A | 449 | ASP | OD1 |
| 1 | A | 449 | ASP | OD2 |
| 1 | A | 449 | ASP | O |
| 1 | A | 451 | ILE | CG2 |
| 1 | A | 451 | ILE | CD1 |
| 1 | A | 451 | ILE | O |
| 1 | A | 452 | ASN | CA |
| 1 | A | 452 | ASN | CB |
| 1 | A | 452 | ASN | CG |
| 1 | A | 452 | ASN | OD1 |
| 1 | A | 452 | ASN | ND2 |
| 1 | A | 452 | ASN | C |
| 1 | A | 452 | ASN | O |
| 1 | A | 453 | LEU | N |
| 1 | A | 453 | LEU | CA |
| 1 | A | 453 | LEU | CB |
| 1 | A | 453 | LEU | CD1 |
| 1 | A | 454 | TYR | CE2 |
| 1 | A | 454 | TYR | CD2 |
| 1 | A | 455 | LYS | CA |
| 1 | A | 455 | LYS | CB |
| 1 | A | 455 | LYS | CG |
| 1 | A | 455 | LYS | CD |
| 1 | A | 455 | LYS | CE |
| 1 | A | 455 | LYS | NZ |
| 1 | A | 455 | LYS | O |
| 1 | A | 456 | ASN | CB |
| 1 | A | 456 | ASN | CG |
| 1 | A | 456 | ASN | OD1 |
| 1 | A | 456 | ASN | ND2 |
| 1 | A | 458 | ARG | CB |
| 1 | A | 458 | ARG | CG |
| 1 | A | 458 | ARG | CD |
| 1 | A | 458 | ARG | NE |
| 1 | A | 458 | ARG | CZ |
| 1 | A | 458 | ARG | NH1 |
| 1 | A | 458 | ARG | NH2 |
| 1 | A | 459 | GLU | CA |
| 1 | A | 459 | GLU | CB |
| 1 | A | 459 | GLU | CG |
| 1 | A | 459 | GLU | CD |
| 1 | A | 459 | GLU | OE1 |
| 1 | A | 459 | GLU | OE2 |
| 1 | A | 459 | GLU | O |
| 1 | A | 462 | VAL | CB |
| 1 | A | 462 | VAL | CG1 |
| 1 | A | 462 | VAL | CG2 |
| 1 | A | 463 | TYR | CD1 |
| 1 | A | 463 | TYR | CE1 |
| 1 | A | 463 | TYR | CZ |
| 1 | A | 463 | TYR | OH |
| 1 | A | 463 | TYR | CE2 |
| 1 | A | 463 | TYR | CD2 |
| 1 | A | 491 | TRP | NE1 |
| 1 | A | 491 | TRP | CZ2 |
| 1 | A | 491 | TRP | CH2 |
| 1 | A | 495 | ASN | CG |
| 1 | A | 495 | ASN | OD1 |
| 1 | A | 495 | ASN | ND2 |
| 1 | A | 499 | TRP | NE1 |
| 1 | A | 499 | TRP | CZ2 |
| 1 | A | 499 | TRP | CH2 |
| 1 | A | 503 | SER | O |
| 1 | A | 504 | ILE | O |
| 1 | A | 505 | SER | CA |
| 1 | A | 505 | SER | CB |
| 1 | A | 505 | SER | OG |
| 1 | A | 505 | SER | C |
| 1 | A | 506 | GLY | N |
| 1 | A | 506 | GLY | CA |
| 1 | A | 507 | ALA | N |
| 1 | A | 513 | GLU | OE1 |
| 1 | A | 539 | ILE | CA |
| 1 | A | 539 | ILE | CG2 |
| 1 | A | 539 | ILE | O |
| 1 | A | 542 | SER | CB |
| 1 | A | 542 | SER | OG |
| 1 | A | 542 | SER | C |
| 1 | A | 542 | SER | O |
| 1 | A | 543 | ASN | CA |
| 1 | A | 543 | ASN | OD1 |
| 1 | A | 545 | MET | O |
| 1 | A | 546 | TYR | CA |
| 1 | A | 546 | TYR | CB |
| 1 | A | 546 | TYR | CD1 |
| 1 | A | 546 | TYR | CE1 |
| 1 | A | 546 | TYR | OH |
| 1 | A | 546 | TYR | O |
| 1 | A | 549 | GLY | CA |
| 1 | A | 549 | GLY | O |
| 1 | A | 550 | GLN | CA |
| 1 | A | 550 | GLN | OE1 |
| 1 | A | 550 | GLN | NE2 |
| 1 | A | 550 | GLN | O |
| 1 | A | 551 | TYR | CA |
| 1 | A | 551 | TYR | CD2 |
| 1 | A | 552 | PRO | CB |
| 1 | A | 552 | PRO | CG |
| 1 | A | 552 | PRO | CD |
| 1 | A | 552 | PRO | C |
| 1 | A | 553 | ARG | N |
| 1 | A | 553 | ARG | CA |
| 1 | A | 553 | ARG | CB |
| 1 | A | 553 | ARG | CG |
| 1 | A | 553 | ARG | CD |
| 1 | A | 556 | ARG | CD |
| 1 | A | 556 | ARG | NE |
| 1 | A | 556 | ARG | CZ |
| 1 | A | 556 | ARG | NH1 |
| 1 | A | 556 | ARG | NH2 |
| 1 | A | 578 | ASP | CA |
| 1 | A | 578 | ASP | OD1 |
| 1 | A | 578 | ASP | OD2 |
| 1 | A | 578 | ASP | O |
| 1 | A | 579 | GLY | CA |
| 1 | A | 581 | GLN | CB |
| 1 | A | 581 | GLN | OE1 |
| 1 | A | 582 | ASP | CA |
| 1 | A | 582 | ASP | CB |
| 1 | A | 582 | ASP | CG |
| 1 | A | 582 | ASP | OD1 |
| 1 | A | 582 | ASP | OD2 |
| 1 | A | 582 | ASP | O |
| 1 | A | 583 | MET | CG |
| 1 | A | 583 | MET | SD |
| 1 | A | 583 | MET | CE |
| 1 | A | 586 | ASP | CA |
| 1 | A | 586 | ASP | CB |
| 1 | A | 586 | ASP | CG |
| 1 | A | 586 | ASP | OD1 |
| 1 | A | 586 | ASP | OD2 |
| 1 | A | 586 | ASP | C |
| 1 | A | 586 | ASP | O |
| 1 | A | 587 | THR | N |
| 1 | A | 587 | THR | CA |
| 1 | A | 587 | THR | CG2 |
| 1 | A | 587 | THR | OG1 |
| 1 | A | 589 | ILE | CB |
| 1 | A | 589 | ILE | CG2 |
| 1 | A | 589 | ILE | CD1 |
| 1 | A | 590 | LYS | N |
| 1 | A | 590 | LYS | CA |
| 1 | A | 590 | LYS | CB |
| 1 | A | 590 | LYS | CG |
| 1 | A | 590 | LYS | CD |
| 1 | A | 590 | LYS | CE |
| 1 | A | 590 | LYS | NZ |
| 1 | A | 593 | GLN | CA |
| 1 | A | 593 | GLN | CB |
| 1 | A | 593 | GLN | CG |
| 1 | A | 593 | GLN | CD |
| 1 | A | 593 | GLN | OE1 |
| 1 | A | 593 | GLN | NE2 |
| 1 | A | 593 | GLN | O |
| 1 | A | 594 | LYS | CA |
| 1 | A | 594 | LYS | CG |
| 1 | A | 594 | LYS | CD |
| 1 | A | 594 | LYS | CE |
| 1 | A | 594 | LYS | NZ |
| 1 | A | 594 | LYS | O |
| 1 | A | 595 | CYS | CA |
| 1 | A | 596 | ARG | N |
| 1 | A | 596 | ARG | CG |
| 1 | A | 596 | ARG | CD |
| 1 | A | 596 | ARG | NE |
| 1 | A | 596 | ARG | CZ |
| 1 | A | 596 | ARG | NH1 |
| 1 | A | 596 | ARG | NH2 |
| 1 | A | 597 | ARG | N |
| 1 | A | 597 | ARG | CB |
| 1 | A | 597 | ARG | CG |
| 1 | A | 597 | ARG | CD |
| 1 | A | 597 | ARG | NE |
| 1 | A | 597 | ARG | NH2 |
| 1 | A | 598 | HIS | CD2 |
| 1 | A | 626 | GLN | CB |
| 1 | A | 626 | GLN | CG |
| 1 | A | 626 | GLN | OE1 |
| 1 | A | 627 | PRO | CB |
| 1 | A | 627 | PRO | CG |
| 1 | A | 627 | PRO | CD |
| 1 | A | 628 | GLN | N |
| 1 | A | 628 | GLN | CA |
| 1 | A | 628 | GLN | CB |
| 1 | A | 628 | GLN | CG |
| 1 | A | 628 | GLN | CD |
| 1 | A | 628 | GLN | OE1 |
| 1 | A | 628 | GLN | NE2 |
| 1 | A | 628 | GLN | O |
| 1 | A | 629 | GLN | CG |
| 1 | A | 629 | GLN | OE1 |
| 1 | A | 629 | GLN | NE2 |
| 1 | A | 631 | HIS | CB |
| 1 | A | 631 | HIS | NE2 |
| 1 | A | 631 | HIS | CD2 |
| 1 | A | 632 | THR | CG2 |
| 1 | A | 632 | THR | OG1 |
| 1 | A | 635 | GLU | CD |
| 1 | A | 635 | GLU | OE1 |
| 1 | A | 635 | GLU | OE2 |
| 1 | A | 639 | TYR | CA |
| 1 | A | 639 | TYR | OH |
| 1 | A | 639 | TYR | CE2 |
| 1 | A | 639 | TYR | CD2 |
| 1 | A | 642 | GLY | CA |
| 1 | A | 642 | GLY | O |
| 1 | A | 643 | ALA | CA |
| 1 | A | 643 | ALA | CB |
| 1 | A | 643 | ALA | C |
| 1 | A | 643 | ALA | O |
| 1 | A | 644 | GLN | O |
| 1 | A | 645 | THR | CG2 |
| 1 | A | 650 | GLN | NE2 |
| 1 | A | 682 | PRO | CB |
| 1 | A | 685 | VAL | CG1 |
| 1 | A | 685 | VAL | O |
| 1 | A | 686 | LYS | CA |
| 1 | A | 686 | LYS | CB |
| 1 | A | 686 | LYS | CG |
| 1 | A | 686 | LYS | CD |
| 1 | A | 686 | LYS | CE |
| 1 | A | 686 | LYS | O |
| 1 | A | 689 | GLY | CA |
| 1 | A | 689 | GLY | C |
| 1 | A | 690 | SER | N |
| 1 | A | 690 | SER | CA |
| 1 | A | 690 | SER | OG |
| 1 | A | 693 | LYS | CA |
| 1 | A | 693 | LYS | CB |
| 1 | A | 693 | LYS | CG |
| 1 | A | 693 | LYS | CD |
| 1 | A | 693 | LYS | CE |
| 1 | A | 693 | LYS | NZ |
| 1 | A | 693 | LYS | O |
| 1 | A | 696 | VAL | CA |
| 1 | A | 696 | VAL | CB |
| 1 | A | 696 | VAL | CG1 |
| 1 | A | 696 | VAL | O |
| 1 | A | 697 | ARG | CG |
| 1 | A | 697 | ARG | CD |
| 1 | A | 697 | ARG | NE |
| 1 | A | 697 | ARG | CZ |
| 1 | A | 697 | ARG | NH1 |
| 1 | A | 697 | ARG | NH2 |
| 1 | A | 699 | CYS | CB |
| 1 | A | 699 | CYS | SG |
| 1 | A | 699 | CYS | O |
| 1 | A | 700 | LYS | N |
| 1 | A | 700 | LYS | CA |
| 1 | A | 700 | LYS | CB |
| 1 | A | 700 | LYS | CG |
| 1 | A | 700 | LYS | CD |
| 1 | A | 700 | LYS | CE |
| 1 | A | 700 | LYS | NZ |
| 1 | A | 700 | LYS | O |
| 1 | A | 701 | ALA | O |
| 1 | A | 702 | VAL | O |
| 1 | A | 703 | GLY | CA |
| 1 | A | 704 | HIS | N |
| 1 | A | 704 | HIS | CB |
| 1 | A | 705 | PRO | CG |
| 1 | A | 705 | PRO | CD |
| 1 | A | 737 | GLU | CB |
| 1 | A | 737 | GLU | CD |
| 1 | A | 737 | GLU | OE1 |
| 1 | A | 737 | GLU | OE2 |
| 1 | A | 737 | GLU | C |
| 1 | A | 737 | GLU | O |
| 1 | A | 738 | MET | N |
| 1 | A | 738 | MET | CA |
| 1 | A | 738 | MET | SD |
| 1 | A | 740 | THR | CB |
| 1 | A | 740 | THR | CG2 |
| 1 | A | 740 | THR | O |
| 1 | A | 741 | LYS | N |
| 1 | A | 741 | LYS | CA |
| 1 | A | 741 | LYS | CB |
| 1 | A | 741 | LYS | CG |
| 1 | A | 741 | LYS | CD |
| 1 | A | 741 | LYS | CE |
| 1 | A | 741 | LYS | NZ |
| 1 | A | 743 | PRO | CA |
| 1 | A | 743 | PRO | CB |
| 1 | A | 743 | PRO | CG |
| 1 | A | 743 | PRO | C |
| 1 | A | 743 | PRO | O |
| 1 | A | 744 | LEU | N |
| 1 | A | 744 | LEU | CA |
| 1 | A | 746 | ARG | CA |
| 1 | A | 746 | ARG | CB |
| 1 | A | 746 | ARG | CG |
| 1 | A | 746 | ARG | CD |
| 1 | A | 746 | ARG | NE |
| 1 | A | 746 | ARG | CZ |
| 1 | A | 746 | ARG | NH1 |
| 1 | A | 746 | ARG | NH2 |
| 1 | A | 746 | ARG | C |
| 1 | A | 746 | ARG | O |
| 1 | A | 747 | SER | N |
| 1 | A | 747 | SER | CA |
| 1 | A | 747 | SER | CB |
| 1 | A | 747 | SER | OG |
| 1 | A | 747 | SER | O |
| 1 | A | 749 | ARG | CB |
| 1 | A | 749 | ARG | CD |
| 1 | A | 749 | ARG | NE |
| 1 | A | 749 | ARG | CZ |
| 1 | A | 749 | ARG | NH1 |
| 1 | A | 749 | ARG | NH2 |
| 1 | A | 750 | THR | CA |
| 1 | A | 750 | THR | CB |
| 1 | A | 750 | THR | CG2 |
| 1 | A | 750 | THR | OG1 |
| 1 | A | 751 | VAL | CG2 |
| 1 | A | 753 | ARG | CB |
| 1 | A | 753 | ARG | CD |
| 1 | A | 753 | ARG | NE |
| 1 | A | 753 | ARG | CZ |
| 1 | A | 753 | ARG | NH1 |
| 1 | A | 753 | ARG | NH2 |
| 1 | A | 754 | GLU | CD |
| 1 | A | 754 | GLU | OE1 |
| 1 | A | 754 | GLU | OE2 |
| 1 | A | 756 | LEU | CB |
| 1 | A | 757 | LYS | N |
| 1 | A | 757 | LYS | CA |
| 1 | A | 757 | LYS | CB |
| 1 | A | 757 | LYS | CG |
| 1 | A | 757 | LYS | CD |
| 1 | A | 757 | LYS | CE |
| 1 | A | 757 | LYS | NZ |
| 1 | A | 757 | LYS | O |
| 1 | A | 758 | LEU | CA |
| 1 | A | 758 | LEU | O |
| 1 | A | 760 | SER | CB |
| 1 | A | 760 | SER | OG |
| 1 | A | 760 | SER | C |
| 1 | A | 761 | GLY | N |
| 1 | A | 761 | GLY | CA |
| 1 | A | 761 | GLY | C |
| 1 | A | 761 | GLY | O |
| 1 | A | 764 | SER | CB |
| 1 | A | 764 | SER | OG |
| 1 | A | 764 | SER | O |
| 1 | A | 765 | ARG | CA |
| 1 | A | 765 | ARG | CB |
| 1 | A | 765 | ARG | CG |
| 1 | A | 765 | ARG | CD |
| 1 | A | 765 | ARG | NE |
| 1 | A | 765 | ARG | CZ |
| 1 | A | 765 | ARG | NH1 |
| 1 | A | 765 | ARG | NH2 |
| 1 | A | 765 | ARG | O |
| 1 | A | 767 | ASN | CB |
| 1 | A | 767 | ASN | ND2 |
| 1 | A | 793 | PRO | O |
| 1 | A | 794 | ALA | CA |
| 1 | A | 794 | ALA | CB |
| 1 | A | 794 | ALA | O |
| 1 | A | 796 | ARG | O |
| 1 | A | 797 | GLU | CD |
| 1 | A | 797 | GLU | OE1 |
| 1 | A | 797 | GLU | OE2 |
| 1 | A | 798 | PRO | CG |
| 1 | A | 798 | PRO | CD |
| 1 | A | 799 | GLU | CG |
| 1 | A | 799 | GLU | CD |
| 1 | A | 799 | GLU | OE1 |
| 1 | A | 799 | GLU | OE2 |
| 1 | A | 802 | SER | CA |
| 1 | A | 802 | SER | CB |
| 1 | A | 802 | SER | OG |
| 1 | A | 802 | SER | C |
| 1 | A | 802 | SER | O |
| 1 | A | 803 | THR | CA |
| 1 | A | 803 | THR | OG1 |
| 1 | A | 806 | THR | CG2 |
| 1 | A | 806 | THR | OG1 |
| 1 | A | 809 | ASN | CB |
| 1 | A | 809 | ASN | CG |
| 1 | A | 809 | ASN | OD1 |
| 1 | A | 809 | ASN | ND2 |
| 1 | A | 809 | ASN | O |
| 1 | A | 810 | LYS | CD |
| 1 | A | 810 | LYS | CE |
| 1 | A | 810 | LYS | NZ |
| 1 | A | 838 | PHE | CB |
| 1 | A | 838 | PHE | CD1 |
| 1 | A | 838 | PHE | CE1 |
| 1 | A | 838 | PHE | CZ |
| 1 | A | 838 | PHE | CE2 |
| 1 | A | 838 | PHE | C |
| 1 | A | 838 | PHE | O |
| 1 | A | 839 | GLU | CA |
| 1 | A | 839 | GLU | CB |
| 1 | A | 839 | GLU | CG |
| 1 | A | 839 | GLU | OE1 |
| 1 | A | 839 | GLU | C |
| 1 | A | 839 | GLU | O |
| 1 | A | 840 | GLU | CA |
| 1 | A | 840 | GLU | CB |
| 1 | A | 840 | GLU | CG |
| 1 | A | 840 | GLU | OE1 |
| 1 | A | 841 | TYR | N |
| 1 | A | 841 | TYR | CA |
| 1 | A | 841 | TYR | CD1 |
| 1 | A | 841 | TYR | CE1 |
| 1 | A | 841 | TYR | CZ |
| 1 | A | 841 | TYR | OH |
| 1 | A | 841 | TYR | CE2 |
| 1 | A | 841 | TYR | CD2 |
| 1 | A | 842 | PRO | CA |
| 1 | A | 842 | PRO | CB |
| 1 | A | 842 | PRO | CG |
| 1 | A | 842 | PRO | CD |
| 1 | A | 842 | PRO | C |
| 1 | A | 842 | PRO | O |
| 1 | A | 843 | GLU | CA |
| 1 | A | 843 | GLU | CB |
| 1 | A | 843 | GLU | CG |
| 1 | A | 843 | GLU | CD |
| 1 | A | 843 | GLU | OE1 |
| 1 | A | 843 | GLU | OE2 |
| 1 | A | 844 | HIS | ND1 |
| 1 | A | 844 | HIS | CE1 |
| 1 | A | 845 | ARG | CD |
| 1 | A | 845 | ARG | NE |
| 1 | A | 846 | THR | CA |
| 1 | A | 846 | THR | CB |
| 1 | A | 846 | THR | CG2 |
| 1 | A | 846 | THR | OG1 |
| 1 | A | 846 | THR | C |
| 1 | A | 846 | THR | O |
| 1 | A | 847 | HIS | CA |
| 1 | A | 847 | HIS | ND1 |
| 1 | A | 847 | HIS | CE1 |
| 1 | A | 847 | HIS | NE2 |
| 1 | A | 849 | PHE | CB |
| 1 | A | 850 | TYR | N |
| 1 | A | 850 | TYR | CB |
| 1 | A | 850 | TYR | CG |
| 1 | A | 850 | TYR | CD1 |
| 1 | A | 850 | TYR | CE1 |
| 1 | A | 850 | TYR | CZ |
| 1 | A | 850 | TYR | OH |
| 1 | A | 850 | TYR | CE2 |
| 1 | A | 850 | TYR | CD2 |
| 1 | A | 853 | GLN | CB |
| 1 | A | 853 | GLN | CG |
| 1 | A | 853 | GLN | CD |
| 1 | A | 853 | GLN | OE1 |
| 1 | A | 853 | GLN | NE2 |
| 1 | A | 854 | ALA | N |
| 1 | A | 854 | ALA | CA |
| 1 | A | 854 | ALA | CB |
| 1 | A | 857 | SER | CB |
| 1 | A | 857 | SER | OG |
| 1 | A | 858 | HIS | NE2 |
| 1 | A | 858 | HIS | CD2 |
| 1 | A | 882 | PHE | O |
| 1 | A | 883 | LYS | O |
| 1 | A | 884 | HIS | CB |
| 1 | A | 884 | HIS | ND1 |
| 1 | A | 884 | HIS | O |
| 1 | A | 885 | THR | N |
| 1 | A | 885 | THR | CA |
| 1 | A | 885 | THR | CB |
| 1 | A | 885 | THR | CG2 |
| 1 | A | 885 | THR | OG1 |
| 1 | A | 885 | THR | C |
| 1 | A | 885 | THR | O |
| 1 | A | 886 | MET | N |
| 1 | A | 886 | MET | CA |
| 1 | A | 886 | MET | CB |
| 1 | A | 886 | MET | CG |
| 1 | A | 886 | MET | SD |
| 1 | A | 886 | MET | CE |
| 1 | A | 887 | ARG | N |
| 1 | A | 887 | ARG | CA |
| 1 | A | 887 | ARG | CB |
| 1 | A | 887 | ARG | CG |
| 1 | A | 887 | ARG | CD |
| 1 | A | 887 | ARG | NE |
| 1 | A | 887 | ARG | CZ |
| 1 | A | 887 | ARG | NH1 |
| 1 | A | 887 | ARG | NH2 |
| 1 | A | 888 | ASN | N |
| 1 | A | 888 | ASN | CA |
| 1 | A | 888 | ASN | CB |
| 1 | A | 888 | ASN | CG |
| 1 | A | 888 | ASN | OD1 |
| 1 | A | 888 | ASN | ND2 |
| 1 | A | 888 | ASN | O |
| 1 | A | 889 | VAL | CA |
| 1 | A | 890 | ALA | CB |
| 1 | A | 891 | ASP | CB |
| 1 | A | 891 | ASP | CG |
| 1 | A | 891 | ASP | OD1 |
| 1 | A | 891 | ASP | OD2 |
| 1 | A | 892 | THR | N |
| 1 | A | 892 | THR | CA |
| 1 | A | 892 | THR | CB |
| 1 | A | 892 | THR | CG2 |
| 1 | A | 892 | THR | OG1 |
| 1 | A | 894 | LEU | CD1 |
| 1 | A | 895 | GLN | CA |
| 1 | A | 895 | GLN | CG |
| 1 | A | 895 | GLN | CD |
| 1 | A | 895 | GLN | OE1 |
| 1 | A | 895 | GLN | NE2 |
| 1 | A | 898 | TYR | CB |
| 1 | A | 898 | TYR | CG |
| 1 | A | 898 | TYR | CD1 |
| 1 | A | 898 | TYR | CE1 |
| 1 | A | 898 | TYR | CZ |
| 1 | A | 898 | TYR | OH |
| 1 | A | 898 | TYR | CE2 |
| 1 | A | 898 | TYR | CD2 |
| 1 | A | 929 | VAL | CG1 |
| 1 | A | 929 | VAL | O |
| 1 | A | 930 | VAL | CA |
| 1 | A | 930 | VAL | CG2 |
| 1 | A | 930 | VAL | O |
| 1 | A | 932 | ASP | O |
| 1 | A | 933 | THR | CA |
| 1 | A | 933 | THR | CB |
| 1 | A | 933 | THR | CG2 |
| 1 | A | 933 | THR | OG1 |
| 1 | A | 933 | THR | C |
| 1 | A | 933 | THR | O |
| 1 | A | 934 | SER | N |
| 1 | A | 934 | SER | CA |
| 1 | A | 934 | SER | CB |
| 1 | A | 934 | SER | OG |
| 1 | A | 934 | SER | O |
| 1 | A | 935 | HIS | N |
| 1 | A | 935 | HIS | CA |
| 1 | A | 935 | HIS | CB |
| 1 | A | 935 | HIS | ND1 |
| 1 | A | 935 | HIS | CE1 |
| 1 | A | 935 | HIS | NE2 |
| 1 | A | 935 | HIS | C |
| 1 | A | 935 | HIS | O |
| 1 | A | 936 | THR | N |
| 1 | A | 936 | THR | CA |
| 1 | A | 936 | THR | CB |
| 1 | A | 936 | THR | CG2 |
| 1 | A | 936 | THR | OG1 |
| 1 | A | 936 | THR | O |
| 1 | A | 937 | ALA | N |
| 1 | A | 937 | ALA | CA |
| 1 | A | 937 | ALA | CB |
| 1 | A | 937 | ALA | O |
| 1 | A | 938 | GLY | N |
| 1 | A | 938 | GLY | CA |
| 1 | A | 938 | GLY | C |
| 1 | A | 938 | GLY | O |
| 1 | A | 939 | LEU | N |
| 1 | A | 939 | LEU | CA |
| 1 | A | 939 | LEU | CB |
| 1 | A | 939 | LEU | CG |
| 1 | A | 939 | LEU | CD1 |
| 1 | A | 940 | THR | N |
| 1 | A | 940 | THR | CB |
| 1 | A | 940 | THR | CG2 |
| 1 | A | 940 | THR | OG1 |
| 1 | A | 941 | MET | N |
| 1 | A | 941 | MET | CG |
| 1 | A | 941 | MET | SD |
| 1 | A | 941 | MET | CE |
| 1 | A | 942 | HIS | CB |
| 1 | A | 944 | SER | CB |
| 1 | A | 944 | SER | OG |
| 1 | A | 988 | HIS | CD2 |
| 1 | A | 988 | HIS | O |
| 1 | A | 989 | LEU | CA |
| 1 | A | 989 | LEU | CD2 |
| 1 | A | 993 | GLN | OE1 |
| 1 | A | 993 | GLN | NE2 |
| 1 | A | 1006 | GLN | O |
| 1 | A | 1007 | ASP | CA |
| 1 | A | 1007 | ASP | OD1 |
| 1 | A | 1007 | ASP | OD2 |
| 1 | A | 1008 | ILE | N |
| 1 | A | 1008 | ILE | CB |
| 1 | A | 1008 | ILE | CG2 |
| 1 | A | 1008 | ILE | CG1 |
| 1 | A | 1008 | ILE | CD1 |
| 1 | A | 1009 | PRO | CA |
| 1 | A | 1009 | PRO | CB |
| 1 | A | 1009 | PRO | CG |
| 1 | A | 1009 | PRO | CD |
| 1 | A | 1009 | PRO | C |
| 1 | A | 1009 | PRO | O |
| 1 | A | 1010 | ALA | N |
| 1 | A | 1010 | ALA | CA |
| 1 | A | 1010 | ALA | CB |
| 1 | A | 1012 | LYS | CB |
| 1 | A | 1012 | LYS | CG |
| 1 | A | 1012 | LYS | CD |
| 1 | A | 1012 | LYS | CE |
| 1 | A | 1012 | LYS | NZ |
| 1 | A | 1013 | GLU | CA |
| 1 | A | 1013 | GLU | CB |
| 1 | A | 1013 | GLU | CG |
| 1 | A | 1013 | GLU | CD |
| 1 | A | 1013 | GLU | OE1 |
| 1 | A | 1013 | GLU | OE2 |
| 1 | A | 1013 | GLU | O |
| 1 | A | 1016 | ARG | CB |
| 1 | A | 1016 | ARG | CG |
| 1 | A | 1016 | ARG | CD |
| 1 | A | 1016 | ARG | NE |
| 1 | A | 1016 | ARG | CZ |
| 1 | A | 1016 | ARG | NH1 |
| 1 | A | 1016 | ARG | NH2 |
| 1 | A | 1016 | ARG | C |
| 1 | A | 1016 | ARG | O |
| 1 | A | 1017 | ASP | CA |
| 1 | A | 1017 | ASP | OD1 |
| 1 | A | 1019 | LEU | CG |
| 1 | A | 1019 | LEU | CD1 |
| 1 | A | 1019 | LEU | CD2 |
| 1 | A | 1020 | VAL | CG1 |
| 1 | A | 1020 | VAL | CG2 |
| 1 | A | 1020 | VAL | O |
| 1 | A | 1021 | GLN | CA |
| 1 | A | 1021 | GLN | CB |
| 1 | A | 1021 | GLN | CG |
| 1 | A | 1021 | GLN | O |
| 1 | A | 1023 | LYS | CB |
| 1 | A | 1023 | LYS | CG |
| 1 | A | 1023 | LYS | CD |
| 1 | A | 1023 | LYS | CE |
| 1 | A | 1023 | LYS | NZ |
| 1 | A | 1023 | LYS | C |
| 1 | A | 1023 | LYS | O |
| 1 | A | 1024 | GLU | CA |
| 1 | A | 1024 | GLU | CB |
| 1 | A | 1024 | GLU | CG |
| 1 | A | 1024 | GLU | CD |
| 1 | A | 1024 | GLU | OE1 |
| 1 | A | 1024 | GLU | OE2 |
| 1 | A | 1024 | GLU | C |
| 1 | A | 1024 | GLU | O |
| 1 | A | 1025 | PHE | C |
| 1 | A | 1025 | PHE | O |
| 1 | A | 1026 | ALA | N |
| 1 | A | 1026 | ALA | C |
| 1 | A | 1026 | ALA | O |
| 1 | A | 1027 | GLY | N |
| 1 | A | 1027 | GLY | CA |
| 1 | A | 1027 | GLY | O |
| 1 | A | 1030 | THR | CB |
| 1 | A | 1030 | THR | OG1 |
| 1 | A | 1031 | SER | N |
| 1 | A | 1031 | SER | CB |
| 1 | A | 1031 | SER | OG |
| 1 | A | 1032 | ASP | N |
| 1 | A | 1032 | ASP | CG |
| 1 | A | 1032 | ASP | OD1 |
| 1 | A | 1032 | ASP | OD2 |
| 1 | A | 1034 | PHE | CB |
| 1 | A | 1034 | PHE | CG |
| 1 | A | 1034 | PHE | CD1 |
| 1 | A | 1034 | PHE | CE1 |
| 1 | A | 1034 | PHE | O |
| 1 | A | 1037 | GLU | CB |
| 1 | A | 1037 | GLU | CG |
| 1 | A | 1037 | GLU | C |
| 1 | A | 1037 | GLU | O |
| 1 | A | 1038 | ARG | CA |
| 1 | A | 1038 | ARG | CG |
| 1 | A | 1041 | SER | CA |
| 1 | A | 1041 | SER | CB |
| 1 | A | 1041 | SER | OG |
| 1 | A | 1041 | SER | C |
| 1 | A | 1041 | SER | O |
| 1 | A | 1042 | LEU | CD2 |
| 1 | A | 1044 | GLN | CB |
| 1 | A | 1044 | GLN | CG |
| 1 | A | 1045 | ALA | N |
| 1 | A | 1045 | ALA | CA |
| 1 | A | 1045 | ALA | CB |
| 1 | A | 1045 | ALA | O |
| 1 | A | 1048 | GLU | CB |
| 1 | A | 1048 | GLU | CG |
| 1 | A | 1048 | GLU | CD |
| 1 | A | 1048 | GLU | OE1 |
| 1 | A | 1048 | GLU | OE2 |
| 1 | A | 1048 | GLU | C |
| 1 | A | 1048 | GLU | O |
| 1 | A | 1049 | LYS | N |
| 1 | A | 1049 | LYS | CA |
| 1 | A | 1049 | LYS | CB |
| 1 | A | 1049 | LYS | CG |
| 1 | A | 1049 | LYS | CD |
| 1 | A | 1049 | LYS | O |
| 1 | A | 1050 | HIS | CE1 |
| 1 | A | 1051 | LYS | N |
| 1 | A | 1051 | LYS | CG |
| 1 | A | 1051 | LYS | CD |
| 1 | A | 1051 | LYS | CE |
| 1 | A | 1051 | LYS | NZ |
| 1 | A | 1052 | ILE | N |
| 1 | A | 1052 | ILE | CB |
| 1 | A | 1052 | ILE | CG2 |
| 1 | A | 1052 | ILE | CG1 |
| 1 | A | 1052 | ILE | CD1 |
| 1 | A | 1053 | GLN | N |
| 1 | A | 1053 | GLN | CA |
| 1 | A | 1053 | GLN | CB |
| 1 | A | 1053 | GLN | CG |
| 1 | A | 1053 | GLN | CD |
| 1 | A | 1053 | GLN | OE1 |
| 1 | A | 1053 | GLN | NE2 |
| 1 | A | 1053 | GLN | C |
| 1 | A | 1053 | GLN | O |
| 1 | A | 1054 | MET | N |
| 1 | A | 1054 | MET | O |
| 1 | A | 1055 | SER | CA |
| 1 | A | 1055 | SER | CB |
| 1 | A | 1056 | VAL | N |
| 1 | A | 1056 | VAL | CB |
| 1 | A | 1056 | VAL | CG1 |
| 1 | A | 1056 | VAL | CG2 |
| 1 | A | 1056 | VAL | O |
| 1 | A | 1058 | GLY | CA |
| 1 | A | 1058 | GLY | C |
| 1 | A | 1058 | GLY | O |
| 1 | A | 1059 | ILE | N |
| 1 | A | 1059 | ILE | CA |
| 1 | A | 1059 | ILE | CB |
| 1 | A | 1059 | ILE | CG2 |
| 1 | A | 1059 | ILE | CG1 |
| 1 | A | 1059 | ILE | CD1 |
| 1 | A | 1059 | ILE | C |
| 1 | A | 1059 | ILE | O |
| 1 | A | 1060 | LEU | CA |
| 1 | A | 1060 | LEU | O |
| 1 | A | 1061 | ASN | N |
| 1 | A | 1061 | ASN | CA |
| 1 | A | 1061 | ASN | CB |
| 1 | A | 1061 | ASN | CG |
| 1 | A | 1061 | ASN | OD1 |
| 1 | A | 1061 | ASN | ND2 |
| 1 | A | 1062 | PRO | CB |
| 1 | A | 1062 | PRO | CG |
| 1 | A | 1062 | PRO | CD |
| 1 | A | 1062 | PRO | C |
| 1 | A | 1062 | PRO | O |
| 1 | A | 1063 | HIS | N |
| 1 | A | 1063 | HIS | CA |
| 1 | A | 1063 | HIS | CB |
| 1 | A | 1063 | HIS | CG |
| 1 | A | 1063 | HIS | ND1 |
| 1 | A | 1063 | HIS | CE1 |
| 1 | A | 1063 | HIS | NE2 |
| 1 | A | 1063 | HIS | CD2 |
| 1 | A | 1063 | HIS | O |
| 1 | A | 1064 | GLU | CG |
| 1 | A | 1064 | GLU | OE1 |
| 1 | A | 1065 | ILE | CG1 |
| 1 | A | 1065 | ILE | CD1 |
| 1 | A | 1066 | PRO | N |
| 1 | A | 1066 | PRO | CA |
| 1 | A | 1066 | PRO | CB |
| 1 | A | 1066 | PRO | CG |
| 1 | A | 1066 | PRO | CD |
| 1 | A | 1066 | PRO | C |
| 1 | A | 1066 | PRO | O |
| 1 | A | 1067 | GLU | CA |
| 1 | A | 1067 | GLU | CB |
| 1 | A | 1067 | GLU | CG |
| 1 | A | 1067 | GLU | CD |
| 1 | A | 1067 | GLU | OE1 |
| 1 | A | 1067 | GLU | OE2 |
| 1 | A | 1067 | GLU | O |
| 1 | A | 1070 | CYS | CB |
| 1 | A | 1070 | CYS | SG |
| 1 | A | 1071 | ASP | CB |
| 1 | A | 1071 | ASP | CG |
| 1 | A | 1071 | ASP | OD1 |
| 1 | A | 1071 | ASP | OD2 |
