# Supplementary material for: Identification of natural antiviral drug candidates against Tilapia Lake Virus: Computational drug design approaches
Source: PLoS One. 2023 Nov 8;18(11):e0287944. doi: 10.1371/journal.pone.0287944 (PMC10631680; doi:10.1371/journal.pone.0287944)
Supplement: S2 Table — These compounds were chosen for their relatively high binding affinities. The table illustrates the compounds, PubChem CID, their chemical names, molecular formulas, molecular weight, and docking scores of the seventeen selected compounds with highest binding affinities. (DOCX) [file pone.0287944.s005.docx]

| **SI No.** | **Pubchem ID** | **Chemical Name** | **Molecular Formula** | **Chemical structure** | **Molecular weight (g/mol)** | **Binding Affinity (Kcal/mol)** |
| --- | --- | --- | --- | --- | --- | --- |
| 1 | CID 107876 | Procyanidin | C30H26O13 | 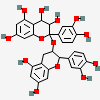 | 594.5 | -8.3 |
| 2 | CID12795736 | delta7-Avenasterol | C29H48O | 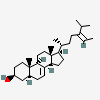 | 412.7 | -8.2 |
| 3 | CID 12303662 | Phytosterols | C29H50O | 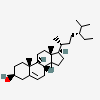 | 414.7 | -7.9 |
| 4 | CID 5997 | Cholesterol | C27H46O | 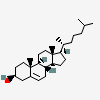 | 386.7 | -7.1 |
| 5 | CID 10134 | phenanthren-3-ol | C28H48O | 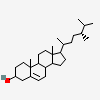 | 400.7 | -7.0 |
| 6 | CID 638072 | squalene | C30H50 | 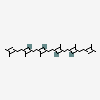 | 410.7 | -7.0 |
| 7 | CID 10448938 | Epiquinine | C20H24N2O2 | 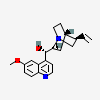 | 324.4 | -7.0 |
| 8 | CID  7280268 | trideca-1(9),2(7)-dien-3-one | C20H25N3O3S | 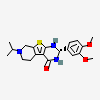 | 387.5 | -6.8 |
| 9 | CID 985 | palmitic acid | C16H32O2 | 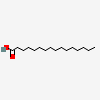 | 256.42 | -6.2 |
| 10 | CID 3026 | dibutyl phthalate | C16H22O4 | 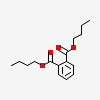 | 278.34 | -6.1 |
| 11 | CID 5634 | undecylenic acid | C11H20O2 | 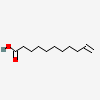 | 184.27 | -6.0 |
| 12 | CID 6009 | aminophenazone | C13H17N3O | 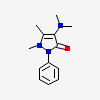 | 231.29 | -5.9 |
| 13 | CID 8181 | Methyl hexadecanoate | C17H34O2 | 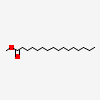 | 270.5 | -5.7 |
| 14 | CID 11006 | n-Hexadecane | C16H34 | 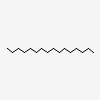 | 226.44 | -5.6 |
| 15 | CID  560653 | Chitaric acid | C6H10O6 | 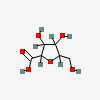 | 178.14 | -5.4 |
| 16 | CID  5163241 | 2-(2-Methylpropyl)cyclohexyl acetate | C12H22O2 | 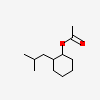 | 198.30 | -5.3 |
| 17 | CID5284421 | Methyl linoleate | C19H34O2 | 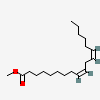 | 294.5 | -5.1 |
