# Supplementary material for: Identification of natural antiviral drug candidates against Tilapia Lake Virus: Computational drug design approaches
Source: PLoS One. 2023 Nov 8;18(11):e0287944. doi: 10.1371/journal.pone.0287944 (PMC10631680; doi:10.1371/journal.pone.0287944)
Supplement: S3 Table — This table illustrates the three compounds selected for their binding affinities and their respective RMSD values analyzed through the process of re-docking. (DOCX) [file pone.0287944.s006.docx]

| Ligand | Binding Affinity | rmsd/ub | rmsd/lb |
| --- | --- | --- | --- |
| CID 107876 | -8.3 | 0 | 0 |
| CID 107876 | -8.3 | 21.156 | 16.154 |
| CID 107876 | -8.2 | 3.765 | 2.364 |
| CID 107876 | -8.2 | 3.021 | 2.064 |
| CID 107876 | -8.2 | 6.333 | 3.127 |
| CID 107876 | -8.2 | 7.322 | 1.805 |
| CID 107876 | -8 | 39.918 | 35.973 |
| CID 107876 | -8 | 19.562 | 14.446 |
| CID 107876 | -7.9 | 8.375 | 1.998 |
| CID 12795736 | -8.2 | 0 | 0 |
| CID 12795736 | -7.7 | 8.744 | 5.061 |
| CID 12795736 | -7.6 | 8.803 | 5.01 |
| CID 12795736 | -7.5 | 8.088 | 4.842 |
| CID 12795736 | -7.2 | 7.237 | 4.398 |
| CID 12795736 | -7 | 5.153 | 3.298 |
| CID 12795736 | -7 | 9.043 | 5.93 |
| CID 12795736 | -6.9 | 7.942 | 5.435 |
| CID 12795736 | -6.8 | 7.147 | 4.638 |
| CID 12303662 | -7.9 | 0 | 0 |
| CID 12303662 | -7.8 | 8.613 | 5.228 |
| CID 12303662 | -7.5 | 7.993 | 4.602 |
| CID 12303662 | -7.4 | 4.096 | 1.804 |
| CID 12303662 | -7.2 | 2.449 | 1.119 |
| CID 12303662 | -7.1 | 52.99 | 49.928 |
| CID 12303662 | -7.1 | 3.005 | 2.415 |
| CID 12303662 | -7.1 | 6.692 | 4.187 |
| CID 12303662 | -7 | 9.927 | 3.366 |
