# Supplementary material for: Identification of natural antiviral drug candidates against Tilapia Lake Virus: Computational drug design approaches
Source: PLoS One. 2023 Nov 8;18(11):e0287944. doi: 10.1371/journal.pone.0287944 (PMC10631680; doi:10.1371/journal.pone.0287944)
Supplement: S4 Table — This is a tabulation of RMS deviations as estimated using FlexX and SeeSAR software. (DOCX) [file pone.0287944.s007.docx]

| rms | total | close, act |
| --- | --- | --- |
| ≤0.5 | 3 | 3,0 |
| 0.5−1.0 | 0 | 0,0 |
| 1.0−1.5 | 1 | 1,0 |
| 1.5−2.0 | 3 | 3,0 |
| 2.0−3.5 | 6 | 4,2 |
| >3.5 | 14 | 10,4 |
