# Supplementary material for: Identification of natural antiviral drug candidates against Tilapia Lake Virus: Computational drug design approaches
Source: PLoS One. 2023 Nov 8;18(11):e0287944. doi: 10.1371/journal.pone.0287944 (PMC10631680; doi:10.1371/journal.pone.0287944)
Supplement: S5 Table — The steric and electrostatic effects arising from the spatial arrangement of three most preferable compounds are tabulated & presented here. (DOCX) [file pone.0287944.s008.docx]

| Name | pAct | MW | HA | HD | LogP | TPSA | RBs | MR | Length | Max Length | Optimal PC | r&sup2; | SDEC | q&sup2; | SDEP | r&sup2; YS |
| --- | --- | --- | --- | --- | --- | --- | --- | --- | --- | --- | --- | --- | --- | --- | --- | --- |
| Structure2D_COMPOUND_CID_107876.GM | 8.222 | 594.137 | 13 | 10 | 2.733 | 229.99 | 14 | 144.305 | 11.931 | 12.795 | * | 1 | 0 | 1 | 0 |  |
| Structure2D_COMPOUND_CID_12303662.GM | 8.222 | 414.386 | 1 | 1 | 8.025 | 20.23 | 13 | 128.217 | 15.727 | 16.627 |  | 1 | 0 | 1 | 0 |  |
| Structure2D_COMPOUND_CID_12795736.GM | 8.222 | 412.371 | 1 | 1 | 7.945 | 20.23 | 12 | 128.193 | 11.354 | 12.939 |  | 1 | 0 | 1 | 0 |  |
