# Supplementary material for: Identification of natural antiviral drug candidates against Tilapia Lake Virus: Computational drug design approaches
Source: PLoS One. 2023 Nov 8;18(11):e0287944. doi: 10.1371/journal.pone.0287944 (PMC10631680; doi:10.1371/journal.pone.0287944)
Supplement: S6 Table — Calculated MM/GBSA (binding free energy) and No Strain (NS) binding values and their variance calculated for each of the selected three compounds. (DOCX) [file pone.0287944.s009.docx]

| **Compound** | **MMGBSA-dG-binding energy** | **MMGBSA-dG-bind in Coulomb** | **MMGBSA-dG-bind(NS)** | **MMGBSA-dG bind(NS)-Coulomb** |
| --- | --- | --- | --- | --- |
| **CID 107876** | -41.8499±  5.08 | -13.9768±  6.54 | -53.0216±  6.48 | -22.1245±  6.39 |
| **CID 12795736** | -49.1706±  12.41 | -19.4305±  12.39 | -62.5148±  12.43 | -19.5074±  11.35 |
| **CID 12303662** | -35.0858±  8.19 | -15.2166±  9.72 | -58.5522±  10.49 | -14.9453±  11.02 |
