# Supplementary material for: Identification of natural antiviral drug candidates against Tilapia Lake Virus: Computational drug design approaches
Source: PLoS One. 2023 Nov 8;18(11):e0287944. doi: 10.1371/journal.pone.0287944 (PMC10631680; doi:10.1371/journal.pone.0287944)
Supplement: Attachment — Submitted filename: Response to reviewers.docx [file pone.0287944.s010.docx]

**Reviewer #1:**

**Comment:** The English need improvement since there are some grammatical and syntax errors in the manuscript. For example, in line number 97, the words “a easy” may be as “an easy”
**Reply:** Thank you for your suggestion. Corrected as you suggested. One of our corresponding authors is from the US is a native English speaker, and has checked the entire manuscript for language, format and proofreading errors.

**Comment:** in line number 113, “in same” as “in the same”;

**Reply:** Corrected as suggested.

**Comment:** in line number 137, “NCBI” as “the NCBI”;

**Reply:** Corrected as recommended.

**Comment:** in line number 141, “anticipated” as “the anticipated”;

**Reply:** Corrected as suggested.

**Comment:** in line number 145, “it in online” as “it online”;

**Reply:** Thank you, corrected as suggested.

**Comment:** in line number 172, “had identified” as “identified”;

**Reply:** Corrected as suggested.

**Comment:** in line number 173, “Lamarckian” as “the Lamarckian”;

**Reply:** Corrected as suggested.

**Comment:** in line number 259, “2D” as “the 2D”;

**Reply:** Corrected as suggested.

**Comment:** in line number 319, “has been” as “have been”;

**Reply:** Corrected as recommended.

**Comment:** in line number 336, “compounds was” as “compounds were”;

**Reply:** Corrected as suggested.

**Comment:** in line number 381, “simulations is” as “simulations are”;

**Reply:** Thanks for catching this error; Corrected as suggested.

**Comment:** in line number 397, “lowest” as “the lowest”;

**Reply:** Corrected as suggested.

**Comment:** in line number 404, “highest” as “the highest”.

**Reply:** Corrected as suggested.

**Comment:** The grammar mistakes which are not mentioned here are also to be checked and corrected properly.

**Reply:** Thank you for your advice and we have rechecked and proofread the final version.

**Comment: 2.** There are some typing mistakes as well, and authors are advised to carefully proof-read the text. For example,
**Comment:** in line number 118, the word “naturally” may be as “natural”;

**Reply:** Please see the response above.

**Comment:** in line number 150, “refine” as “refined”;

**Reply:** Corrected as suggested.

**Comment:** in line number 158, “plants” as “plant”;

**Reply:** Revised as suggested.

**Comment:** in line number 247, “Protein‐ Ligand” as “Protein‐Ligand”;

**Reply:** This error has been corrected.

**Comment:** Table 3, “Pharmakokinetics” as “Pharmacokinetics”;

**Reply:** Spelling has been corrected as suggested.

**Comment:** in line number 334, “shown” as “show”;

**Reply:** Corrected as suggested.

**Comment:** in line number 340, “dynamic” as “dynamics”;

**Reply:** Corrected as suggested.

**Comment:** in line number 398, “refine” as “refined”;

**Reply:** Corrected as suggested.

**Comment:** in line number 421, “cost effective” as “cost-effective”.

**Reply:** OK, revised as suggested.

**Comment:** The typos not mentioned here are also to be checked and corrected properly.

**Reply:** Agreed; the revised manuscript has been carefully proofread.

**Comment: 3.** Check the abbreviations throughout the manuscript and introduce the abbreviation when the full word appears the first time in the abstract and the remaining for the text and then use only the abbreviation (For example, molecular dynamics (MD), CRM1, etc.,). Make a word abbreviated in the article that is repeated at least three times in the text, not all words to be abbreviated. The usage of abbreviations may be avoided in the keywords.

**Reply:** Thank you for your advice and acronyms have been used as recommended.

**Comment: 4.** The author should uniformly follow some terms, for example, either “in silico” or “in- silico”.

**Reply:** Thank you for your crucial suggestion; corrected for uniformity.

**Comment: 5.** The figure legends should be improved and a proper footnote should be given. All legends should have enough description for a reader to understand the figures without having to refer back to the main text of the manuscript. For example, the necessary abbreviations should be given.

**Reply:** Thank you for your suggestion and included footnote where needed for figures and tables.

**Comment: 6.** The conclusion seems in general. All conclusions must be convincing statements on what was found to be novel, impact based on the strong support of the data/results/discussion. Moreover, the authors may also be included the limitation of the present findings for a better understanding of the manuscript.

**Reply:** Thank you for this suggestion; we have revised to address your concern.

**Reviewer #2:** Authors of the manuscript entitled “Identification of Natural Antiviral Drug Candidates Against Tilapia Lake Virus: Computational Drug Design Approaches” introduced computational approach for investigating the anti-viral potentiality for natural metabolites isolated from *Heritiera fomes* and *Ceriops candolleana*. Molecular docking analysis, in silico ADMET analysis, and molecular dynamics studies have highlighted the promising molecular reactivity and binding affinity of three identified hits. The manuscript is valuable in its field as it redeems publication following the address of these suggestions and comments:

**Comment: 1.** The structure, nomenclature, and physiochemical properties of the identified 17 compounds should be presented even within the supplementary materials.

**Reply:** Thank you for your suggestion. These were added in the supplementary materials, Table S2.

**Comment: 2.** Section 3.2. Phytochemical and Protein Preparation, should be relocated to the materials and methods section rather than the in Results part.

**Reply:** We shifted it to the materials and methods section as suggested.

**Comment: 3.** Ramachandran plot should be presented at the supplementary materials.

**Reply:** Ramachandran plot is compiled in the supplementary file, see Figure 2.

**Comment: 4.** The authors should adopt a positive control throughout the computational analysis to assess how the obtained results for the isolated natural metabolites would be of biological significance. Positive reference control could be a reported or market-approved inhibitor for the adopted CRM1 biotarget.

**Reply:** Thank you for your insightful comment. We completely agree with your suggestion to incorporate a positive control in our computational analysis to evaluate the biological significance of the isolated natural metabolites. Including a positive reference control, e.g. a reported or market-approved inhibitor for the adopted CRM1 biotarget, would greatly enhance the robustness and reliability of our findings. We used Cα as a positive control. We appreciate your valuable input, and make sure to include a positive control in the studies. This additional analysis helped to further validate the relevance and potential therapeutic implications of the identified natural metabolites. Thank you again for your suggestion, and we are committed to addressing this issue to improve the quality and comprehensiveness of our research.

**Comment: 5.** Validation of the docking protocol should be done through redocking protocol using the same docking algorism and parameters that are adopted for the investigated compounds has been recognized as a well-reported approach (See References; doi: 10.1021/jm0302997; doi: 10.1016/j.ejps.2020.105510; doi: 10.1016/j.bmcl.2019.02.031; doi: 10.1016/j.bioorg.2022.105770; doi: 10.3390/biology10050389). The ability of the molecular modelling simulation to replicate the ligand binding mode and residue-wise interaction patterns with low RMSD values (< 2.0 Å) between the native its redocked pose ensure the validity of the adopted protocol to provide binding pose of biological significance.

**Reply:** Thank you for your suggestion. We followed your advice and ran the redocking procedure again. Please see the table S3.

**Comment: 6.** Authors provided comparative data regarding ligand binding modes through both highlighted polar hydrogen bonds and hydrophobic contacts. However, hydrogen binding should be presented within hydrogen bond distances as well as bond angles since hydrogen bond depend on both. Authors should mention the Hydrogen bond angles as well as their distances, since the strength of hydrogen bonding is based on both parameters in a way to ensure the adequacy of optimum hydrogen bonding.

**Reply:** Thank you for your thoughtful feedback on our manuscript. We acknowledge the importance of presenting comprehensive data related to hydrogen bonding in our ligand binding analysis. In our revised manuscript, we will provide both hydrogen bond distances and angles to offer a more complete understanding of the interactions. We agree that hydrogen bond strength depends not only on the distance but also on the bond angle. By considering both parameters, we can ensure the adequacy of optimum hydrogen bonding and provide a more accurate evaluation of ligand binding modes. We greatly appreciate your suggestion, as it will enhance the clarity and rigor of our research. Your feedback is instrumental in improving the quality of our work, and we ensured to incorporate this information in revisions. Please see table 2 and table S5.

**Comment: 7.** Line 49, docking scores should be presented within their respective Kcal/mol units.

**Reply:** Docking scores have been presented as suggested above.

**Comment: 8.** Through the RMSD and RMSF analysis, authors should illustrate trajectories for apo protein as well. This approach would better highlight the impact of compound’s binding on target through pinpointing flexible and immobile patterns for the protein ternary structures and amino acids in reference to the unliganded form. Difference RMSF (ΔRMSF = RMSFApo-Holo) could be adopted as well (please refer to doi: 10.1016/j.bmcl.2019.02.031 and doi: 10.1016/j.bmcl.2019.02.031).

**Reply:** Thank you for your insightful comment regarding our manuscript. We appreciate your suggestion to include trajectories for the apo protein in addition to the compound-bound complexes. We agree that incorporating RMSD (Root-Mean-Square Deviation) and RMSF (Root-Mean-Square Fluctuation) analysis of the apo protein would provide a more comprehensive understanding of the impact of the compound's binding on the target. By comparing the ternary structures of the protein and identifying flexible and immobile patterns, we can effectively highlight the conformational changes induced by the ligand binding. We ensured that these trajectories are included in our revised manuscript; see Figure 8. This addition will certainly enhance the clarity and significance of our findings. We sincerely appreciate your feedback and constructive input. Thank you for your suggestions.

**Comment: 9.** Authors are advised to overlay of the initial, middle, and final frames (at 0 ns, 50 ns, and 100 ns, respectively) for each ligand-protein complex across the molecular dynamics simulations. This approach would provide great insights regarding the time-evolution orientation/conformation changes for both the protein and bounded ligands as well as the conserved and reformed ligand-amino acid bindings and close-range contacts.

**Reply:** Rearranged as suggested; please see Figure 6 and Figure 7.

**Comment: 10.** Findings from the MM-GBSA free binding energy calculations as well as the constituting energy terms (VDW, electrostatic, solvation, SASA) should be represented in Figure or Table for better understanding the nature of ligand-target binding and the main driving energy terms for future structural improvements/optimization.

**Reply:** Thank you for your valuable feedback regarding the representation of the MM-GBSA free binding energy calculations and energy terms in our manuscript. We appreciate the suggestion to include Figures 10 and Table S6 to better understand the nature of ligand-target binding and identify the main driving energy terms for future structural improvements/optimizations. We agree that a visual representation of the MM-GBSA free binding energy calculations and the constituent energy terms (VDW, electrostatic, solvation, SASA) would enhance the clarity and comprehensibility of our findings. By presenting these data in a Figure 11, readers will have a clearer understanding of the contributing energy terms and their relative importance. Per your suggestion, we have revised our manuscript to include a table summarizing the MM-GBSA free binding energy calculations and the individual energy terms associated with each ligand-target complex. Table S5 and S6 provides a concise overview of the binding energies and allows for easier comparison among different ligands. Additionally, we have included a figure that visualizes the contributions of the VDW, electrostatic, solvation, and SASA energy terms for each ligand. This figure offers a clear representation of the main driving energy terms. Thanks sincerely; these modifications significantly improve the manuscript's readability and facilitate a better understanding of the ligand-target binding interactions and energy contributions.

**Comment 11:** Authors should elaborate more on the discussion section through presenting comparative findings from reported literature studies that investigated other natural-isolated and/or even structural-related metabolites against the same target protein.

**Reply:** Thank you for your constructive feedback regarding the elaboration of our discussion section in relation to findings from previous literature studies. We appreciate your suggestion to include comparative findings from studies that investigated other natural-isolated metabolites or structurally-related compounds against the CRM1 target protein. We understand the need for a comprehensive discussion that considers related studies in the field. By including comparative findings highlights the novelty and significance of our work while giving context regarding previous research. We have expanded our discussion to incorporate a comprehensive review of relevant investigations of naturally-isolated metabolites or structurally-related compounds targeting the CRM1 protein. We have analyzed and compared our findings with those of these studies, highlighting similarities, differences, and potential areas of further exploration. This strengthens the discussion and provides valuable insights into the potential implications and applications of our research. We appreciate your thoughtful guidance in improving the quality of our manuscript.

**Comment: 12.** Finally, within the discussion sections, authors should highlight the takeaway messages that would be adopted in future lead optimization and development base on the docking, MD simulations, and ADMET studies. particularly, since two natural metabolites disobey Lipinski’s rule and cannot be considered as relevant clinical candidates. Prospective/recommended structure modifications to improve the ligand’s binding and interactions, as well as pharmacokinetics should be provided within the discussion and conclusion sections.

**Reply:** Thank you for your insightful feedback regarding our manuscript's discussion of future lead optimization and development based on docking, MD simulations, and ADMET studies. We appreciate your suggestion to highlight the potential modifications and improvements that could be made to the ligand's binding, interactions, and pharmacokinetics for future development. We agree that discussing prospective structure modifications and their potential impact on enhancing the ligand's properties is essential for guiding future lead optimization. Also, explicitly addressing the fact that two natural metabolites disobey Lipinski's rule and cannot be considered relevant clinical candidates is important to provide clarity on our findings. As suggested, we have revised our discussion and conclusion sections to identify the limitations of the two natural metabolites based on Lipinski's rule violation. We have also highlighted the need for further modifications to improve the ligand's binding interactions and pharmacokinetic profile. Furthermore, we have provided detailed recommendations for prospective structure modifications that could enhance ligand binding affinity, selectivity, and pharmacokinetics. These modifications take into account the insights gained from our docking, MD simulations, and ADMET studies. By including these recommendations, we not only address the limitations of the current compounds but also provide a roadmap for future lead optimization efforts. Thanks for these improvements based on your valuable feedback.

**Reviewer #3:**

**Comment 1.** Authors should discuss about the current clinical or preclinical or discovery status of CRM1 inhibitors as antiviral agents.

**Reply:** Thank you for your valuable feedback on our manuscript. We appreciate your suggestion to discuss the current clinical, preclinical, or discovery status of CRM1 inhibitors as antiviral agents. The revised manuscript includes a new section specifically addressing the current status of CRM1 inhibitors as antiviral agents. Specifically, we highlight their potential of CRM1 inhibitors as therapeutic agents targeting viral replication and discuss the challenges and opportunities in this area of research. We appreciate your guidance; this addition greatly enhances the relevance and comprehensiveness of our manuscript.

**Comment: 2.** Is the selected target CRM1 validated clinically for viral inhibition??

**Reply:** Thank you for your valuable input; your query regarding the clinical validation of CRM1 as a target for viral inhibition is appreciated. Currently, clinical validation of CRM1 as a target for viral inhibition is limited although several preclinical studies have demonstrated the potential of CRM1 inhibitors as antiviral agents, showing promising results in inhibiting viral replication and reducing viral load in in vitro and animal models of viral infections. Furthermore, some recent clinical trials have explored the use of CRM1 inhibitors in the treatment of certain viral infections, although these trials are ongoing or in the early stages. These trials aim to evaluate the safety, tolerability, and efficacy of CRM1 inhibitors as antiviral agents in human subjects. In our revised manuscript, we have provided a comprehensive discussion on the current clinical and preclinical status of CRM1 inhibitors as antiviral agents. We acknowledge the limited clinical validation of this target but highlight the potential it holds based on promising preclinical data and ongoing clinical trials. We hope that this adequately addresses your concern. We appreciate your valuable feedback and ensure that this information is accurately conveyed in our revised manuscript.

**Comment: 3.** Section 3.3- During the simulation procedure of molecular docking, the server-identified binding sites were used to build a receptor grid with grid box dimensions. Did the authors have generated grid box covering all the four pockets shown in Figure 1?

**Reply:** Thank you for your valuable feedback; your inquiry regarding the coverage of grid boxes in molecular docking simulations to ensure proper inclusion of all the four pockets reflected in Figure 1. For clarification we have explained the grid box dimensions and coverage in our manuscript. In our docking simulations, we utilized the receptor grid generation feature provided in the molecular docking software. We utilized the server-identified binding sites to generate the receptor grid. However, we acknowledge that we did not explicitly mention the coverage of the grid box dimensions for each pocket in our initial manuscript. Per your suggestion, we have revised our manuscript to include a more detailed regarding grid box dimensions and the coverage of all four pockets (see Figure 1). We have now provided clear information on how the grid boxes were placed to encompass each pocket adequately. This allows for more accurate representation and inclusion of all the important binding sites in our simulations. We appreciate your thorough review and constructive feedback, which have helped enhance the clarity and completeness of our manuscript; we believe that our revisions address this concern effectively.

**Comment: 4.** Section 3.4- top 20% of the 17 phytochemicals with the highest binding affinity were selected- How authors have come to this conclusion?? Is docking score alone sufficient to rank the compounds and that to on a predicted target protein and active pocket?? No comparison with standard or with binding interactions.

**Reply:** We appreciate your valuable feedback and concerns raised regarding the selection of the top 20% phytochemicals based on binding affinity in Section 3.4 of our manuscript. We understand your point regarding the limitations of relying solely on docking scores to rank compounds, especially when considering a predicted target protein and active pocket without any reference standards or binding interaction comparisons. We agree that docking scores alone may not be a sufficient basis for definitive conclusions about the binding affinities of the phytochemicals. To address this concern, we will incorporate additional analysis and information to support the selection of the top 20% compounds. Firstly, we will include a comparative analysis of the docking scores with reference standards (e.g. known inhibitors or ligands as available). This will allow for a more reliable ranking and assessment of the binding affinities of the phytochemicals in the context of known compounds. Furthermore, we will also emphasize any observed binding interactions or structural features of the selected compounds among the top 20%, to provide additional support for their inclusion based on potential favorable interactions with active site residues. Per your suggestions, we aim to provide a more comprehensive and meaningful ranking of the phytochemicals by considering both docking scores and comparative analyses with reference standards or binding interactions.

**Comment: 5.** Have to perform MM-PBSA binding energy calculations to observe the energy variations and secondary structure analysis during the simulation time.

**Reply:** We wish to express our gratitude for your valuable input and suggestion regarding the incorporation of MM-PB/GBSA binding energy calculations and secondary structure analysis in our manuscript. We acknowledge the significance of these analyses for observing energy variations and gaining insights into the stability and conformational changes of the system over the simulation time. Consequently, we believe that incorporating MM-GBSA calculations and secondary structure analysis will add depth to our study. To address this matter, we will perform MM-GBSA calculations to evaluate the binding energies of the selected compounds. This analysis will enable us to investigate the stability and energetics of the protein-ligand complexes and rank them accordingly, in order to gain a more comprehensive understanding of the binding affinities. Additionally, we will conduct secondary structure analysis throughout the simulation duration to assess the conformational changes and stability of the protein. This analysis will help us link the changes in secondary structure to the binding interactions and the potential effect on ligand binding. Thank you for highlighting these important aspects, which contributes to the robustness and completeness of our study.
