# Supplementary material for: Identification of natural antiviral drug candidates against Tilapia Lake Virus: Computational drug design approaches
Source: PLoS One. 2023 Nov 8;18(11):e0287944. doi: 10.1371/journal.pone.0287944 (PMC10631680; doi:10.1371/journal.pone.0287944)
Supplement: Attachment — Submitted filename: Review Rebuttal.docx [file pone.0287944.s011.docx]

Please use the space provided to explain your answers to the questions above. You may also include additional comments for the author, including concerns about dual publication, research ethics, or publication ethics. (Please upload your review as an attachment if it exceeds 20,000 characters)

Reviewer #1: 1.

Reviewer #2: The authors addressed almost all suggestions and comments. However, two points should be addressed appropriately:

**Comment 1:** Adopting the protein's Cα as a reference for the analysed MD simulated trajectories is what meant by positive control for the in silico studies. owing to the lack of relevant experimental (wet lab) data, validation of the biological significance for the simulated ligands at docking and MD studies should be performed. Performing the same computational procedures for a literature reported or market-approved drug with experimental activity on the CRM1 biotarget and comparing them with those obtained for the isolated compounds would be relevant to extrapolate these computational data. Thus, it is advised to explore the docking and MD simulation of a literature reported or market-approved drug with CRM1 inhibition activity and compare these data wit those of the identified compounds.

**Reply:** Thank you for your valuable feedback on our research manuscript. We appreciate your insightful comments and suggestions for improving the robustness and relevance of our computational studies on CRM1 inhibition. We completely agree with your suggestion regarding the use of a positive control in our in silico studies. Adopting the Cα of the protein as a reference point for analyzing the MD simulated trajectories is an excellent idea, and we will incorporate this into our methodology to enhance the quality of our research. Furthermore, your recommendation to validate the biological significance of the simulated ligands through experimental validation is well-taken. To address this concern, we plan to extend our study by performing docking and MD simulations on a literature-reported or market-approved drug (Leptomycin B) with known CRM1 inhibition activity. This will allow us to compare our computational findings with existing experimental data, providing a stronger basis for the biological relevance of our results. We appreciate your guidance in enhancing the rigor of our research and ensuring that our computational findings align with experimental observations. Your feedback will undoubtedly strengthen the overall quality and impact of our study. Your expertise in this area is highly valued, and we are committed to addressing all your recommendations to the best of our abilities. Once again, thank you for your time and thoughtful feedback.

**Comment 2:** The authors did not comprehend the concept of overlaying the 3D representation of the ligand-CRM1 complexes at initial, middle, and final frames. Trajectories at 0 ns, 50 ns, and 100 ns should be extracted for each ligand-CRM1 complex and then aligned. This would provide great insights regarding the time-evolution orientation/conformation changes for both the protein and bounded ligands as well as the conserved and reformed ligand-amino acid bindings and close-range contacts. authors are advised to refer to https://doi.org/10.3390/metabo13080942 for further guidance.

**Reply:** Thank you for your feedback and for pointing out the need for a more comprehensive analysis of the ligand-CRM1 complexes in our study. We appreciate your suggestion to overlay the 3D representation of these complexes at different time points throughout the simulation to gain insights into their time-evolution orientation and conformation changes. We understand the importance of this analysis and agree that it would provide a more in-depth understanding of the dynamics of the ligand-protein interactions. Specifically, your recommendation to extract trajectories at 0 ns, 50 ns, and 100 ns for each ligand-CRM1 complex and align them is a valuable approach to explore conserved and reformed ligand-amino acid bindings and close-range contacts over the course of the simulation. We have taken your recommendation seriously and implemented this approach in our analysis (Figure S4). Additionally, we now refer to the resource you provided (https://doi.org/10.3390/metabo13080942) for further guidance on conducting this type of analysis effectively and as a references. Your feedback has been instrumental in enhancing the depth and quality of our research, and we are committed to ensuring that our methodology and analysis align with the best practices in the field. We appreciate your time and expertise in reviewing our work and will work diligently to address your recommendations. Your insights are highly valuable to us, and we are dedicated to meeting the standards of excellence in our research. Thank you once again for your constructive feedback.
